# Synthesis of aryl cyclopropyl sulfides through copper-promoted S-cyclopropylation of thiophenols using cyclopropylboronic acid

**DOI:** 10.3762/bjoc.15.113

**Published:** 2019-05-27

**Authors:** Emeline Benoit, Ahmed Fnaiche, Alexandre Gagnon

**Affiliations:** 1 Département de chimie, Université du Québec à Montréal, C.P. 8888, Succursale Centre-Ville, Montréal, Québec, H3C 3P8, Canada

**Keywords:** aryl cyclopropyl sulfides, copper(II) acetate, copper catalysis, cyclopropylboronic acid, thiophenols

## Abstract

The copper-promoted S-cyclopropylation of thiophenols using cyclopropylboronic acid is reported. The procedure operates under simple conditions to afford the corresponding aryl cyclopropyl sulfides in moderate to excellent yields. The reaction tolerates substitution in *ortho-*, *meta-* and *para*-substitution as well as electron-donating and electron-withdrawing groups. The S-cyclopropylation of a thiophenol was also accomplished using potassium cyclopropyl trifluoroborate.

## Introduction

Aryl cyclopropyl sulfides are present in many biologically active compounds, mainly in their oxidized forms. For example, aryl cyclopropyl sulfones have been used in the preparation of glucokinase (GK) activators for the treatment of type 2 diabetes [1–5] while aryl cyclopropyl sulfoximines have been utilized for the synthesis of modulators of glucokinase regulatory protein (GKRP) [6–8]. Roniciclib, also named BAY 1000394, is a pan-cyclin-dependant kinase (CDK) inhibitor that contains an aryl cyclopropyl sulfoximine and that was developed to treat patients with untreated small cell lung cancer [6,9].

**Scheme 1:**
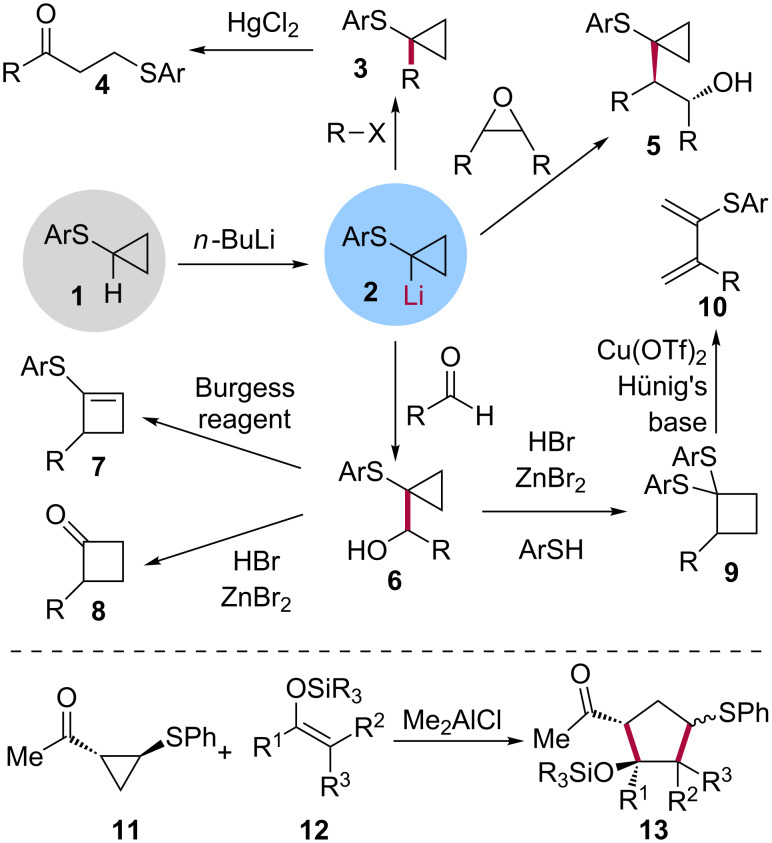
Synthetic uses of aryl cyclopropyl sulfides **1**.

Aryl cyclopropyl sulfides **1** are also remarkable synthons in organic synthesis (Scheme 1). For instance, the proton alpha to the sulfur can be removed by a strong base such as butyllithium, resulting in the cyclopropyllithium species **2**. This carbanion can then react with alkyl halides to provide the corresponding alkylated species **3** which can then be opened up by treatment with mercuric chloride to give the corresponding β-thioaryl ketone **4** [10]. Reacting **2** with epoxides results in the formation of the 1-(β-hydroxy)cyclopropyl aryl sulfides **5** [10] while reaction with formaldehydes [11] or aldehydes [12] affords 1-(arylthio)cyclopropylcarbinyl alcohols **6**. Treating **6** with Burgess reagent or with hydrobromic acid and zinc bromide leads to 1-arylthiocyclobutenes **7** [13] and 2-alkyl-substituted cyclobutanones **8** [11–12], respectively. Treatment of **6** with hydrobromic acid and zinc bromide in the presence of a thiophenol provides the 1,1-di(arylthio)cyclobutane **9** which, upon reaction with copper(II) triflate and Hünig's base, rearranges to give the corresponding 2-(arylthio)-3-alkyl-1,3-butadiene **10** [12]. Reacting methyl 2-phenylthiocyclopropyl ketone **11** with silyl enol ethers **12** in the presence of dimethylaluminium chloride leads to the functionalized cyclopentanes **13** via a highly diastereoselective [3 + 2] cycloaddition reaction [14–15]. The ring expansion sequence **1** → **2** → **6** → **8** has been used as a key step in the synthesis of (±)-fragranol [16], (±)-grandisol [16], (±)-α-cuparenone [17] and (±)-herbertene [17].

**Scheme 2:**
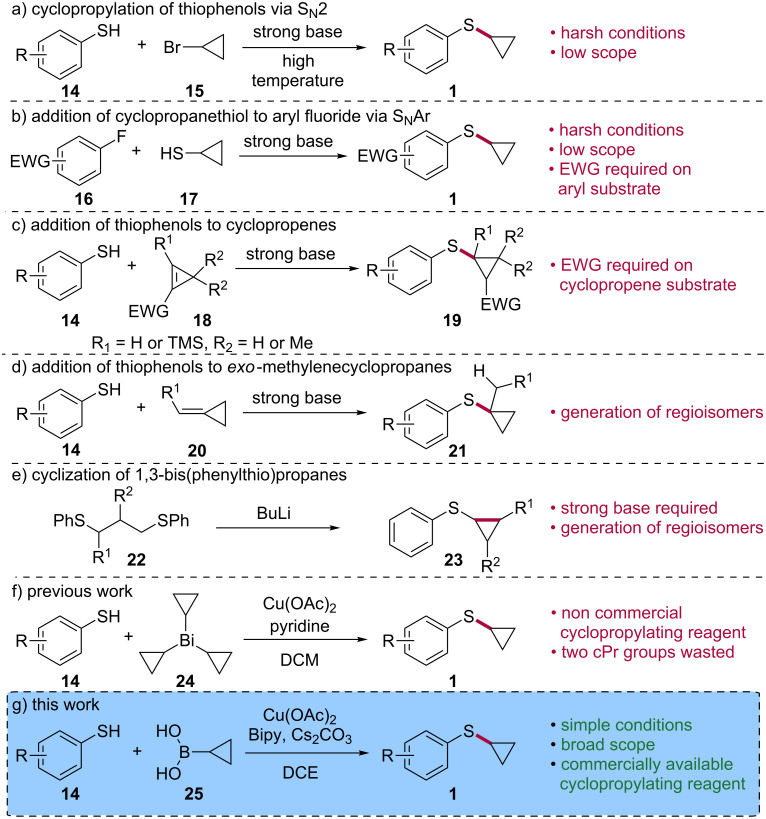
Synthesis of aryl cyclopropyl sulfides.

Aryl cycloropyl sulfides **1** are most frequently prepared by cyclopropylation of thiophenols **14** through S_N_2 reaction with cyclopropyl bromide (**15**, Scheme 2a) [2,4] or by S_N_Ar reaction between aryl fluorides **16** and cyclopropanethiol (**17**, (Scheme 2b) [6]. Although simple and attractive, these approaches usually require harsh conditions such as the presence of a strong base and high temperatures [18]. In addition, an electron-withdrawing group (EWG) must be present on the aryl fluoride **16** for the S_N_Ar reaction to proceed. Aryl cyclopropyl sulfides can also be accessed by the addition of thiophenols **14** to cyclopropenes **18** (Scheme 2c) [19–20] or to *exo*-methylenecyclopropanes **20** (Scheme 2d) [21–22]. While these methods give access to highly substituted products, the requirement for a strong base could jeopardize their application in the context of synthesis of complex molecules. Furthermore, an electron-withdrawing group must be present on **18** to enable the Michael addition with thiol **14**. Treatment of 1,3-bis(phenylthio)propanes **22** with butyllithium is another way of accessing substituted aryl cyclopropyl sulfides **23** (Scheme 2e) [23]. However, in addition to requiring a very strong base, the generation of regio- and stereoisomers from a complex starting material reduces the attractiveness of this method, particularly with respect to medicinal chemistry where expedient methods from easily accessible substrates are needed.

Organobismuth compounds are organometallic reagents that possess a C–Bi bond and which can be synthesized from inexpensive and low-toxic bismuth salts [24–25]. Due to the borderline behavior of bismuth as a metal and a ligand, organobismuth species have been used as reagents and catalysts in a wide range of reactions. We reported a portfolio of methods for the construction of C–C [26–29], C–N [30] and C–O bonds [31–33] using triaryl- and trialkylbismuthines [34]. We also disclosed for the first time in 2007 the synthesis of tricyclopropylbismuth (**24**) and its use in N-cyclopropylation [35], palladium-catalyzed cross coupling [36] and carbonylative cross-coupling reactions [37]. Recently, we demonstrated that tricyclopropylbismuth (**24**) can be used to S-cyclopropylate thiophenols **14**, giving access to aryl cyclopropyl sulfides **1** (Scheme 2f) [38]. While this constituted the first example on the use of an organobismuth reagent in the construction of C(sp^3^)–S bonds, synthetically, the method showed limitations such as the need for a high excess of tricyclopropylbismuth (**24**) which transfers only one cyclopropyl unit out of three to deliver the desired products in moderate yields.

Cyclopropylboronic acid has been elegantly used by Neuville and Zhu [39–40], Tsuritani [41], Taillefer [42], Hayashi [43] and Reddy [44] as a cyclopropylating reagent in N-cyclopropylation reactions, a transformation which is similar to the Chan [45], Evans [46], Lam [47] arylation reaction of N–H and O–H containing substrates. These seminal reports greatly contributed to the synthesis of cyclopropylated compounds in addition to expanding the scope of copper-catalyzed reactions in organic synthesis [48–51]. Our interest in cyclopropylation reactions led us to explore the use of cyclopropylboronic acid in the O-cyclopropylation of phenols. Unfortunately, efficient conditions could not be identified to perform this seemingly simple extension of the N-cyclopropylation reaction. Very recently, Engle and McAlpine disclosed a solution to this problem by developing a highly efficient protocol for the direct O-cyclopropylation of phenols using potassium cyclopropyl trifluoroborate [52]. Surprisingly, and to the best of our knowledge, cyclopropylboronic acid or its various ester and potassium trifluoroborate derivatives have never been used in S-cyclopropylation reactions. In light of the relevance of aryl cyclopropyl sulfides in medicinal and synthetic organic chemistry, we initiated a program to explore the use of cyclopropylboronic acid (**25**) as an S-cyclopropylating agent of thiophenols (Scheme 2g). The publication of copper-catalyzed methods by Feng and Xu to S-arylate thiophenols [53] and by Guy to S-arylate alkyl thiols [54] gave us confidence to proceed ahead with our endeavour for which we herein report our results.

## Results and Discussion

We began by testing the feasibility of S-cyclopropylating 4-*tert*-butylbenzenethiol (**14a**) with cyclopropylboronic acid (**25**) using reaction conditions developed by Neuville and Zhu for the N-cyclopropylation of anilines and amines [39]. Treating thiophenol **14a** with 2.0 equivalents of cyclopropylboronic acid (**25**), 1.0 equivalent of copper(II) acetate, 1.0 equivalent of bipyridine, and 2.0 equivalents of sodium carbonate in dichloroethane at 70 °C for 16 hours provided the desired *S*-cyclopropylated compound **1a** in 86% yield accompanied by only 4% of the diaryl disulfide side-product **26a** (Table 1, entry 1, "standard conditions"). Reducing the catalyst loading by a factor of two under oxygen atmosphere led to a dramatic reduction in the yield of the reaction (Table 1, entry 2). Performing the reaction under oxygen with a stoichiometric amount of copper(II) acetate also proved unsuccessful and afforded mainly the disulfide product, suggesting a deleterious effect of oxygen (Table 1, entry 3). Yet, to our surprise, performing the reaction under argon also negatively impacted the yield of the reaction (Table 1, entry 4), showing that air is the ideal (and also most convenient) atmosphere for this reaction. Changing the solvent for toluene, dichloromethane, dimethylformamide or DMF/H_2_O (4:1) led to lower yields of the desired aryl cyclopropyl sulfide **1a** (Table 1, entry 5) while decreasing the temperature to 50 °C almost completely shut down the reaction (Table 1, entry 6). 1,10-Phenanthroline was found to be the only viable alternative to bipyridine (Table 1, entry 7), with other ligands commonly used in copper-catalyzed reactions such as proline and 2,2,6,6-tetramethyl-3,5-heptanedione giving yields under 15%. Reducing the number of equivalents of boronic acid **25** and sodium carbonate was found to be well tolerated, giving a comparable yield as the "standard conditions" (Table 1, entry 8). While changing the inorganic base to potassium phosphate tribasic or potassium carbonate gave yields below 75%, we found that cesium carbonate provided a net increase in the yield of the reaction (Table 1, entry 9). Attempts at reducing the reaction time led to a minor erosion in the yield of the reaction (Table 1, entry 10). Replacing cyclopropylboronic acid (**25**) with cyclopropylboronic acid pinacol ester (**27**) or cyclopropylboronic acid MIDA ester **28** afforded 85% and 96% of the corresponding diaryl disulfide **26a**, respectively, with no obsevable traces of the desired S-cyclopropylated product **1a** (Table 1, entries 11 and 12). Interestingly, however, potassium cyclopropyl trifluoroborate (**29**) provided the desired aryl cyclopropyl sulfide **1a** in 23% yield, albeit with 30% of the diaryl disulfide side-product **26a** (Table 1, entry 13). Although encouraging, it was clear that the S-cyclopropylation with cPrBF_3_K (**29**) would necessitate extensive optimization and therefore, we decided to pursue our work with cPrB(OH)_2_ (**25**).

**Table 1:** Optimization of the reaction conditions for the copper-promoted S-cyclopropylation of thiophenol **14a** with boron-based cyclopropylating reagents.

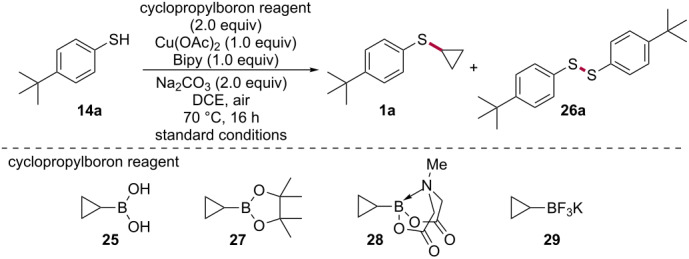			

Entry	Change from "standard conditions"^a^	Yield **1a** (%)^b^	Yield **26a** (%)^b^

1	no change^a^	86	4
2	0.5 equiv of Cu(OAc)_2_ instead of 1.0 equiv under O_2_ instead of air	10	0
3	O_2_ instead of air	19	62
4	argon instead of air	45	4
5	toluene, DCM, DMF or DMF/H_2_O (4:1) instead of DCE	<40	0
6	50 °C instead of 70 °C	7	20
7	1,10-phenanthroline instead of bipy	81	0
8	1.5 equiv of cPrB(OH)_2_ (**25**) instead of 2.0 and 1.0 equiv of Na_2_CO_3_	85	14
**9**	**1.5 equiv of cPrB(OH)_2_ (25) instead of 2.0 and 1.0 equiv of Cs_2_CO_3_ instead of 2.0 equiv of Na_2_CO_3_**	**92**	**6**
10	1.5 equiv of cPrB(OH)_2_ (**25**) instead of 2.0, 1.0 equiv of Cs_2_CO_3_ instead of 2.0 equiv of Na_2_CO_3_ and 6 h instead of 16 h	85	14
11	**27** instead of cPrB(OH)_2_ (**25**)	0	85^c^
12	**28** instead of cPrB(OH)_2_ (**25**)	0	96^c^
13	**29** instead of cPrB(OH)_2_ (**25**)	23	30

^a^Standard conditions: 4-*tert*-butylbenzenethiol (**14a**, 1.0 equiv), cyclopropylboronic acid (**25**, 2.0 equiv), Cu(OAc)_2_ (1.0 equiv), bipyridine (1.0 equiv), Na_2_CO_3_ (2.0 equiv), dichloroethane (0.1 M), 70 °C, 16 h, air. ^b^Yields of isolated pure products. ^c^Conversion calculated by NMR.

**Scheme 3:**
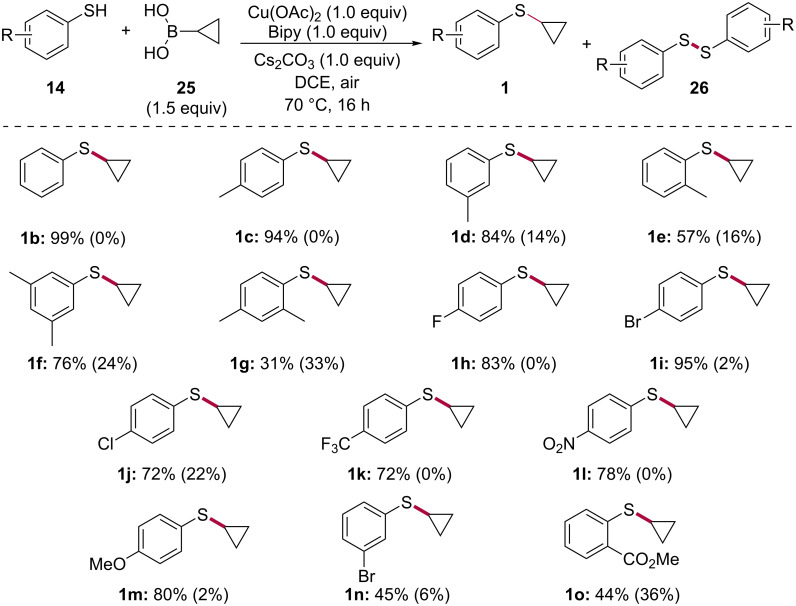
Substrate scope in the copper-promoted S-cyclopropylation of thiophenols **14** using cyclopropylboronic acid (**25**). Numbers in parentheses indicate the yield of isolated pure diaryl disulfide side-products **26**.

With our optimized reactions conditions in hand (i.e., Table 1, entry 9), we embarked on exploring the scope of the copper-promoted S-cyclopropylation of thiophenols using cyclopropylboronic acid (**25**, Scheme 3). Our studies showed that the reaction can be performed on unsubstituted benzenethiol as well as on *para-* and *meta*-methylbenzenethiols, affording the corresponding products **1b**–**d** in 84 to 99% yield. Substitution of the aryl ring at the *ortho*-position resulted in a considerable drop in the efficiency of the process, as indicated by compound **1e** which was obtained in a moderate 57% yield. In line with those results, cyclopropyl(3,5-dimethylphenyl)sulfane (**1f**) was obtained in 76% yield while the 2,4-isomer **1g** was produced in a low 31% yield. Electron-withdrawing groups such as a fluorine, bromine, chlorine, trifluoromethyl and a nitro group as well as electron-donating groups such as a methoxy group at the *para-*position were found to be well tolerated, as indicated by aryl cyclopropyl sulfides **1h**–**m** which were obtained in yields ranging from 72 to 95%. Moving the bromine from the *para-* to the *meta*-position resulted in a substantial reduction in the yield of the reaction, as shown by compound **1n**. Compound **1o** possessing a methyl ester at the *ortho*-position was prepared in 44%, a yield which is consistent with the one obtained for the *ortho*-methyl product **1e**. Compound **1o** indicates some level of tolerance towards functional groups that can be used à posteriori to modify the product. Diaryl disulfides **26** were isolated in yields ranging from 2 to 36%, depending on the thiophenol. Attempts at S-cyclopropylating benzyl mercaptan, an alkylthiol, failed to deliver the desired product.

**Scheme 4:**
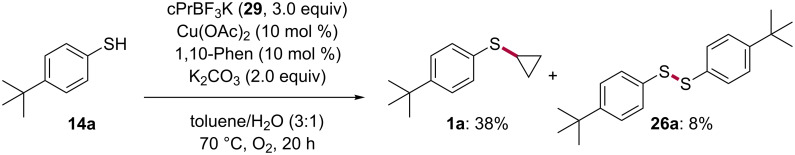
Copper-catalyzed S-cyclopropylation of 4-*tert*-butylbenzenethiol (**14a**) using potassium cyclopropyl trifluoborate (**29**).

Engle and McAlpine recently reported a very efficient, simple and general protocol for the O-cyclopropylation of phenols using potassium cyclopropyl trifluoroborate (**29**) that leads to the corresponding aryl cyclopropyl ethers in good to excellent yields [52]. We wanted to study the transposibility of these conditions to the S-cyclopropylation of thiophenols. In the event, treating 4-*tert*-butylbenzenethiol (**14a**) with 3.0 equivalents of potassium cyclopropyl trifluoroborate (**29**) in the presence of 0.1 equivalents of copper(II) acetate, 0.1 equivalents of 1,10-phenanthroline, 2.0 equivalents of potassium carbonate under oxygen atmosphere at 70 °C for 20 hours in a 3:1 mixture of toluene and water afforded the aryl cyclopropyl sulfide **1a** in 38% along with 8% of the corresponding side-product **26a** and 24% of recovered starting material **14a** (Scheme 4). These results are encouraging and demonstrate that the Engle/McAlpine conditions are applicable, to some extent, to the S-cyclopropylation of thiophenols. It is reasonable to believe that thorough optimization of the reaction conditions should result in a more efficient process. Efforts towards this goal are in progress in our laboratory and results will be reported in due course.

## Conclusion

In conclusion, we developed a simple protocol for the S-cyclopropylation of thiophenols using cyclopropylboronic acid. The reaction is promoted by copper(II) acetate and tolerates electron-withdrawing and electron-donating groups at the *ortho-*, *meta-*, and *para*-positions of the aryl ring to afford the corresponding aryl cyclopropyl sulfides in moderate to excellent yields. This protocol provides an efficient alternative to our previously reported method for the S-cyclopropylation of thiophenols using tricyclopropylbismuth.

## Experimental

### General information

Unless otherwise indicated, all reactions were run under argon in flame-dried glassware. Commercial reagents were used without further purification. Cu(OAc)_2_ (97%) was purchased from Strem Chemicals. Anhydrous solvents were obtained using an encapsulated solvent purification system and were further dried over 4 Å molecular sieves. The evolution of reactions was monitored by analytical thin-layer chromatography using silica gel 60 F254 precoated plates. Flash chromatography was performed employing 230–400 mesh silica using the indicated solvent system according to standard techniques. Proton nuclear magnetic resonance spectra were recorded on a 300 or 600 MHz spectrometer. Chemical shifts for ^1^H NMR spectra are recorded in parts per million from tetramethylsilane with the solvent resonance as the internal standard (chloroform, δ 7.26 ppm). Data is reported as follows: chemical shift δ, multiplicity (s = singlet, d = doublet, t = triplet, dd = doublet of doublet, ddd = doublet of doublet of doublet, td = triplet of doublet, m = multiplet), coupling constant *J* in Hz and integration. Melting points are uncorrected.

### General procedure for the synthesis of aryl cyclopropyl sulfides

A sealed tube equipped with a magnetic stirring bar was charged under ambiant air with cyclopropylboronic acid (**25**, 0.6 mmol, 1.5 equiv), cesium carbonate (0.4 mmol, 1.0 equiv), Cu(OAc)_2_ (0.4 mmol, 1.0 equiv), 2,2'-bipyridine (0.4 mmol, 1.0 equiv) and thiophenol **14** (0.4 mmol, 1.0 equiv). Dichloroethane (0.1 M) was added, the tube was sealed and heated at 70 °C for 16 hours. The reaction mixture was cooled to room temperature and aqueous NH_4_OH 25% (5 mL) was added. The reaction mixture was stirred for a few minutes, transferred in a separatory funnel and extracted with DCM (3 × 5 mL). The combined organic layers were washed with brine (2 × 10 mL), dried over anhydrous Na_2_SO_4_ and concentrated under reduced pressure. The residue was purified by flash column chromatography using the indicated solvent system to afford the corresponding aryl cyclopropyl sulfide **1** and diaryl disulfide **26** as a side-product.

**(4-(*tert*-Butyl)phenyl)(cyclopropyl)sulfane (1a) and 1,2-bis(4-(*tert*-butyl)phenyl)disulfane (26a).** The general procedure was followed on 0.425 mmol scale starting from 4-(*tert-*butyl)benzenethiol (**14a**). The residue was purified on silica gel (100% Hex) to afford **1a** (80.4 mg, 92%) and **26a** (4.2 mg, 6%) as a colorless oil and a white solid, respectively. **1a**: Spectral data was identical to literature compound [38]. ^1^H NMR (300 MHz, CDCl_3_) δ 7.33 (s, 4H), 2.23–2.16 (m, 1H), 1.33 (s, 9H), 1.08–1.02 (m, 2H), 0.73-0.68 (m, 2H). **26a**: mp 65.0–68.5 °C. Spectral data was identical to literature compound [55]. ^1^H NMR (300 MHz, CDCl_3_) δ 7.44 (d, *J* = 8.7 Hz, 2H), 7.33 (d, *J* = 8.4 Hz, 2H), 1.30 (s, 9H).

**Cyclopropyl(phenyl)sulfane (1b).** The general procedure was followed on 0.400 mmol scale starting from benzenethiol (**14b**). The residue was purified on silica gel (100% Hex) to afford **1b** (59.2 mg, 99%) as a slightly yellow oil: Spectral data was identical to literature compound [38]. ^1^H NMR (300 MHz, CDCl_3_) δ 7.39–7.35 (m, 2H), 7.32–7.28 (m, 1H), 7.17–7.11 (m, 1H), 2.23–2.15 (tt, *J* = 8.4, 1.2 Hz, 1H), 1.10–1.04 (m, 2H), 0.72–0.62 (m, 2H).

**Cyclopropyl(*p*-tolyl)sulfane (1c).** The general procedure was followed on 0.400 mmol scale starting from 4-methylbenzenethiol (**14c**). The residue was purified on silica gel (100% Hex) to afford **1c** (61.8 mg, 94%) as a colorless oil: Spectral data was identical to literature compound [38]. ^1^H NMR (300 MHz, CDCl_3_) δ 7.29 (d, *J* = 3.9 Hz, 2H), 7.12 (d, *J* = 4.2 Hz, 2H), 2.33 (s, 3H), 2.22–2.18 (m, 1H), 1.06–1.03 (m, 2H), 0.71–0.68 (m, 2H).

**Cyclopropyl(*m*-tolyl)sulfane (1d) and 1,2-di(*m*-tolyl)disulfane (26d).** The general procedure was followed on 0.400 mmol scale starting from 3-methylbenzenethiol (**14d**). The residue was purified on silica gel (100% Hex) to afford **1d** (55.2 mg, 84%) and **26d** (6.9 mg, 14%) as a colorless and a yellow oil, respectively. **1d**: Spectral data was identical to literature compound [38]. ^1^H NMR (300 MHz, CDCl_3_) δ 7.19–7.18 (m, 3H), 6.97–6.94 (m, 1H), 2.34 (s, 3H), 2.23–2.15 (m, 1H), 1.09–1.03 (m, 2H), 0.72–0.67 (m, 2H). **26d**: Spectral data was identical to literature compound [56]. ^1^H NMR (300 MHz, CDCl_3_) δ 7.32 (s, 2H), 7.30 (d, *J* = 3.9 Hz, 2H), 7.19 (t, *J* = 8.0 Hz, 2H), 7.03 (d, *J* = 7.5 Hz, 2H), 2.32 (s, 6H).

**Cyclopropyl(*o*-tolyl)sulfane (1e) and 1,2-di(*o*-tolyl)disulfane (26e).** The general procedure was followed on 0.400 mmol scale starting from 2-methylbenzenethiol (**14e**). The residue was purified on silica gel (100% Hex) to afford **1e** (37.5 mg, 57%) and **26e** (7.9 mg, 16%) as a slightly yellow and a yellow oil, respectively. **1e**: Spectral data was identical to literature compound [38]. ^1^H NMR (300 MHz, CDCl_3_) δ 7.53 (d, *J* = 7.5 Hz, 1H), 7.20 (td, *J* = 7.5, 1.8 Hz, 1H), 7.15–7.03 (m, 2H), 2.27 (s, 3H), 2.17–2.09 (m, 1H), 1.13–1.07 (m, 2H), 0.73–0.67 (m, 2H). **26e**: Spectral data was identical to literature compound [57]. ^1^H NMR (300 MHz, CDCl_3_) δ 7.52–7.49 (m, 2H), 7.17–7.09 (m, 6H), 2.43 (s, 6H).

**Cyclopropyl(3,5-dimethylphenyl)sulfane (1f) and 1,2-bis(3,5-dimethylphenyl)disulfane (26f).** The general procedure was followed on 0.400 mmol scale starting from 3,5-dimethylbenzenethiol (**14f**). The residue was purified on silica gel (100% Hex) to afford **1f** (54.2 mg, 76%) and **26f** (13.2 mg, 24%) as a colorless and a yellow oil, respectively. **1f**: Spectral data was identical to literature compound [38]. ^1^H NMR (300 MHz, CDCl_3_) δ 6.99 (s, 2H), 6.78 (s, 1H), 2.30 (s, 6H), 2.22–2.14 (m, 1H), 1.08–1.02 (m, 2H), 0.71–0.66 (m, 2H). **26f**: Spectral data was identical to literature compound [58]. ^1^H NMR (300 MHz, CDCl_3_) δ 7.12 (s, 4H), 6.85 (s, 2H), 2.28 (s, 12H).

**Cyclopropyl(2,4-dimethylphenyl)sulfane (1g) and 1,2-bis(2,4-dimethylphenyl)disulfane (26g).** The general procedure was followed on 0.371 mmol scale starting from 2,4-dimethylbenzenethiol (**14g**). The residue was purified on silica gel (100% Hex) to afford **1g** (20.5 mg, 31%) and **26g** (17.3 mg, 33%) as colorless oils. **1g**: Spectral data was identical to literature compound [38]. ^1^H NMR (300 MHz, CDCl_3_) δ 7.41 (d, *J* = 3.9 Hz, 1H), 7.00 (d, *J* = 3.9 Hz, 1H), 6.96 (s, 1H), 2.29 (s, 3H), 2.25 (s, 3H), 2.14–2.10 (m, 1H), 1.07–1.04 (m, 2H), 0.69–0.66 (m, 2H). **26g**: Spectral data was identical to literature compound [38]. ^1^H NMR (300 MHz, CDCl_3_) δ 7.37 (d, *J* = 7.8 Hz, 2H), 6.99 (s, 2H), 6.93 (d, *J* = 8.4 Hz, 2H), 2.37 (s, 6H), 2.29 (s, 6H).

**(4-Fluorophenyl)(cyclopropyl)sulfane (1h).** The general procedure was followed on 0.470 mmol scale starting from 4-fluorobenzenethiol (**14h**). The residue was purified on silica gel (100% Hex) to afford **1h** (65.4 mg, 83%) as a colorless oil: Spectral data was identical to literature compound [38]. ^1^H NMR (300 MHz, CDCl_3_) δ 7.37–7.31 (m, 2H), 7.04–6.96 (m, 2H), 2.22–2.14 (m, 1H), 1.07–1.01 (m, 2H), 0.71–0.66 (m, 2H).

**(4-Bromophenyl)(cyclopropyl)sulfane (1i) and 1,2-bis(4-bromophenyl)disulfane (26i).** The general procedure was followed on 0.400 mmol scale starting from 4-bromobenzenethiol (**14i**). The residue was purified on silica gel (100% Hex) to afford **1i** (86.9 mg, 95%) and **26i** (1.5 mg, 2%) as colorless oils. **1i**: Spectral data was identical to literature compound [38]. ^1^H NMR (300 MHz, CDCl_3_) δ 7.39 (d, *J* = 8.4 Hz, 2H), 7.22 (d, *J* = 8.4 Hz, 2H), 2.20–2.12 (m, 1H), 1.11–1.04 (m, 2H), 0.71–0.66 (m, 2H). **26i**: Spectral data was identical to literature compound [59]. ^1^H NMR (300 MHz, CDCl_3_) δ 7.43 (d, *J* = 8.4, 4H), 7.34 (d, *J* = 8.4 Hz, 4H).

**(4-Chlorophenyl)(cyclopropyl)sulfane (1j) and 1,2-bis(4-chlorophenyl)disulfane (26j).** The general procedure was followed on 0.400 mmol scale starting from 4-chlorobenzenethiol (**14j**). The residue was purified on silica gel (100% Hex) to afford **1j** (52.9 mg, 72%) and **26j** (12.6 mg, 22%) as a colorless oil and a white solid, respectively. **1j**: Spectral data was identical to literature compound [38]. ^1^H NMR (300 MHz, CDCl_3_) δ 7.30–7.22 (m, 4H), 2.20–2.12 (m, 1H), 1.10–1.03 (m, 2H), 0.70–0.65 (m, 2H). **26j**: mp 71.0–73.0 °C. Spectral data was identical to literature compound [60]. ^1^H NMR (300 MHz, CDCl_3_) δ 7.39 (d, *J* = 8.4 Hz, 4H), 7.27 (d, *J* = 8.7, 4H).

**Cyclopropyl(4-(trifluoromethyl)phenyl)sulfane (1k).** The general procedure was followed on 0.400 mmol scale starting from 4-(trifluoromethyl)benzenethiol (**14k**). The residue was purified on silica gel (100% Hex) to afford **1k** (63.2 mg, 72%) as a light yellow oil: Spectral data was identical to literature compound [38]. ^1^H NMR (300 MHz, CDCl_3_) δ 7.52 (d, *J* = 8.4 Hz, 2H), 7.43 (d, *J* = 8.4 Hz, 2H), 2.24–2.15 (m, 1H), 1.18–1.07 (m, 2H), 0.74–0.65 (m, 2H).

**Cyclopropyl(4-nitrophenyl)sulfane (1l).** The general procedure was followed on 0.400 mmol scale starting from 4-nitrobenzenethiol (**14l**). The residue was purified on silica gel (from 100% Hex to 15% EtOAc/Hex) to afford **1l** (60.7 mg, 78%) as a yellow oil: Spectral data was identical to literature compound [38]. ^1^H NMR (300 MHz, CDCl_3_) δ 8.13 (d, *J* = 8.7 Hz, 2H), 7.44 (d, *J* = 9.0 Hz, 2H), 2.25–2.17 (m, 1H), 1.23–1.16 (m, 2H), 0.77–0.72 (m, 2H).

**Cyclopropyl(4-methoxyphenyl)sulfane (1m) and 1,2-bis(4-methoxyphenyl)disulfane (26m).** The general procedure was followed on 0.400 mmol scale starting from 4-methoxybenzenethiol (**14m**). The residue was purified on silica gel (from 100% Hex to 20% EtOAc/Hex) to afford **1m** (57.4 mg, 80%) and **26m** (1.1 mg, 2%) as yellow oils. **1m**: Spectral data was identical to literature compound [38]. ^1^H NMR (300 MHz, CDCl_3_) δ 7.34 (d, *J* = 9.0 Hz, 2H), 6.86 (d, *J* = 8.7 Hz, 2H), 3.80 (s, 3H), 2.22–2.14 (m, 1H), 1.01–0.95 (m, 2H), 0.70–0.65 (m, 2H). **26m**: Spectral data was identical to literature compound [61]. ^1^H NMR (300 MHz, CDCl_3_) δ 7.40 (d, *J* = 9.0 Hz, 4H), 6.83 (d, *J* = 8.7 Hz, 4H), 3.80 (s, 6H).

**(3-Bromophenyl)(cyclopropyl)sulfane (1n) and 1,2-bis(3-bromophenyl)disulfane (26n).** The general procedure was followed on 0.484 mmol scale starting from 3-bromobenzenethiol (**14n**). The residue was purified on silica gel (100% Hex) to afford **1n** (49.9 mg, 45%) and **26n** (5.5 mg, 6%) as a colorless and a yellow oil, respectively. **1n**: Spectral data was identical to literature compound [38]. ^1^H NMR (300 MHz, CDCl_3_) δ 7.51–7.50 (t, *J* = 0.9 Hz, 1H), 7.27–7.24 (m, 2H), 7.13 (t, *J* = 3.9 Hz, 1H), 2.19–2.15 (m, 1H), 1.12–1.09 (m, 2H), 0.72–0.69 (m, 2H). **26n**: Spectral data was identical to literature compound [38]. ^1^H NMR (300 MHz, CDCl_3_) δ 7.64–7.62 (m, 2H), 7.42–7.35 (m, 4H), 7.18 (t, *J* = 7.8 Hz, 2H).

**Methyl 2-(cyclopropylthio)benzoate (1o) and dimethyl 2,2'-disulfanediyldibenzoate (26o).** The general procedure was followed on 0.400 mmol scale starting from methyl 2-mercaptobenzoate (**14o**). The residue was purified on silica gel (from 100% Hex to 20% EtOAC/Hex) to afford **1o** (36.7 mg, 44%) and **26o** (24.1 mg, 36%) as a yellow oil and a white solid, respectively. **1o**: Spectral data was identical to literature compound [38]. ^1^H NMR (300 MHz, CDCl_3_) δ 7.99 (dd, *J* = 8.0, 1.7 Hz, 1H), 7.79 (dd, *J* = 8.1, 1.2 Hz, 1H), 7.47 (ddd, *J* = 8.7, 7.2, 1.5 Hz, 1H), 7.15 (ddd, *J* = 9.0, 7.2, 1.2 Hz, 1H), 3.89 (s, 3H), 2.12–2.04 (m, 1H), 1.17–1.10 (m, 2H), 0.74–0.69 (m, 2H). **26o**: mp 133.0–135.5 °C. Spectral data was identical to literature compound [62]. ^1^H NMR (300 MHz, CDCl_3_) δ 8.06 (dd, *J* = 7.8, 1.5 Hz, 2H), 7.76 (dd, *J* = 8.3, 1.1 Hz, 2H), 7.41 (ddd, *J* = 8.3, 7.3, 1.3 Hz, 2H), 7.23 (dd, *J* = 7.5, 1.2, 2H), 3.99 (s, 6H).

## Supporting Information

File 1 Copies of NMR spectra of synthesized compounds.
