# Supplementary material for: Synthesis of aryl cyclopropyl sulfides through copper-promoted S-cyclopropylation of thiophenols using cyclopropylboronic acid
Source: Beilstein J Org Chem. 2019 May 27;15:1162–71. doi: 10.3762/bjoc.15.113 (PMC6604732; doi:10.3762/bjoc.15.113)
Supplement: File 1 — Copies of NMR spectra of synthesized compounds. [file Beilstein_J_Org_Chem-15-1162-s001.pdf]

BEILSTEIN JOURNAL OF ORGANIC CHEMISTRY

Supporting Information

for

Synthesis of aryl cyclopropyl sulfides through copper-promoted S-cyclopropylation of thiophenols using cyclopropylboronic acid

Emeline Benoit, Ahmed Fnaiche and Alexandre Gagnon

Beilstein J. Org. Chem. **2019,** *15,* 1162–1171. doi:10.3762/bjoc.15.113

Copies of NMR spectra of synthesized compounds

License and Terms: This is a supporting information file under the terms of the Creative Commons Attribution License (http://creativecommons.org/licenses/by/4.0). Please note that the reuse, redistribution and reproduction in particular requires that the authors and source are credited.
The license is subject to the *Beilstein Journal of Organic Chemistry* terms and conditions: (https://www.beilstein-journals.org/bjoc)

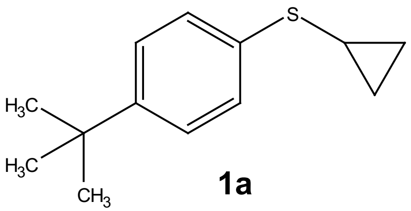

1a

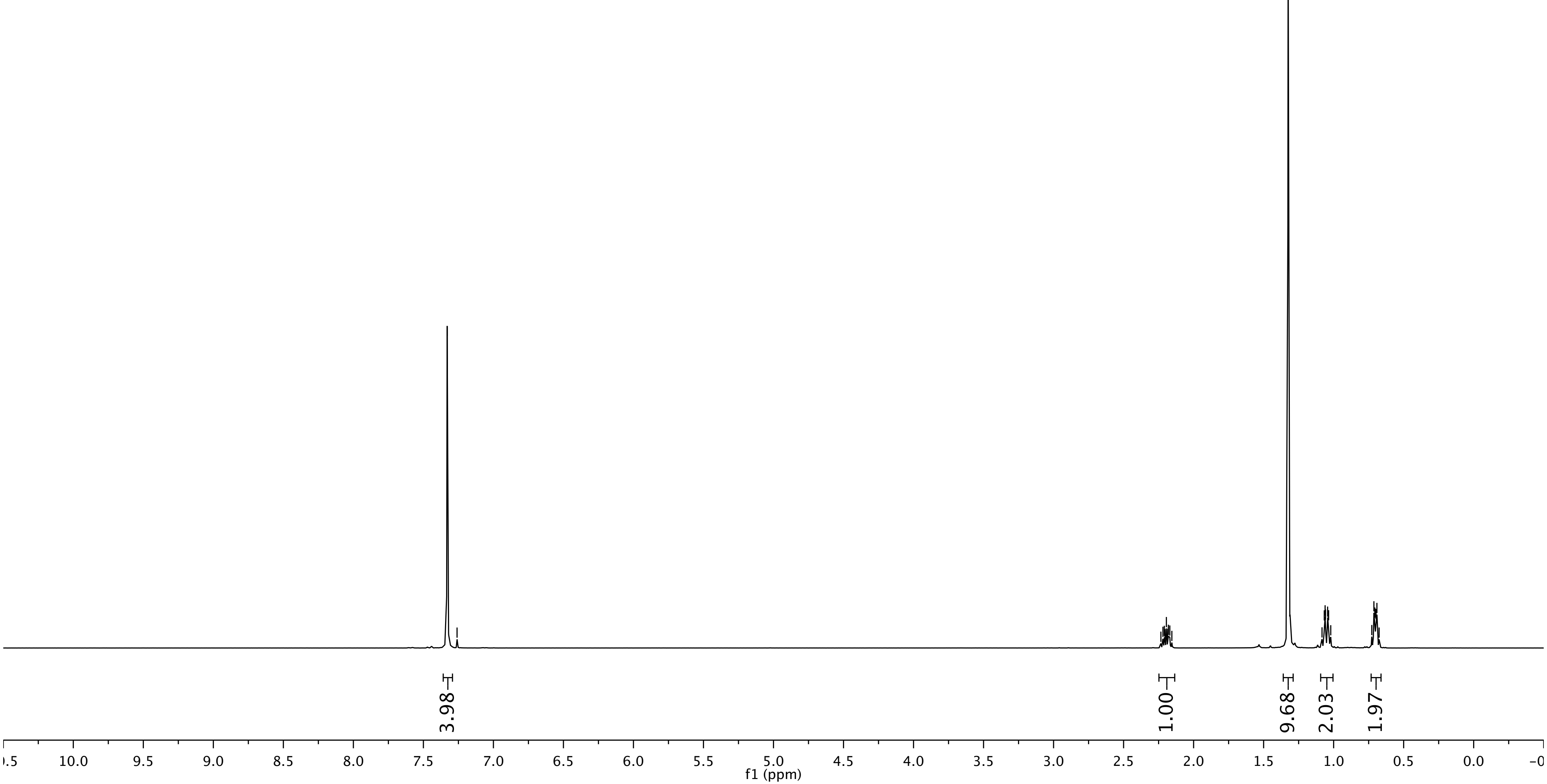

7.330
7.260
2.234
2.219
2.209
2.205
2.194
2.185
2.180
2.170
2.155
1.325
1.083
1.067
1.061
1.058
1.043
1.036
1.021
0.728
0.712
0.706
0.698
0.691
0.675
3.98
1.00
9.68
2.03
1.97
10.0
9.5
9.0
8.5
8.0
7.5
7.0
6.5
6.0
5.5
5.0
4.5
4.0
3.5
3.0
2.5
2.0
1.5
1.0
0.5
0.0
f1 (ppm)

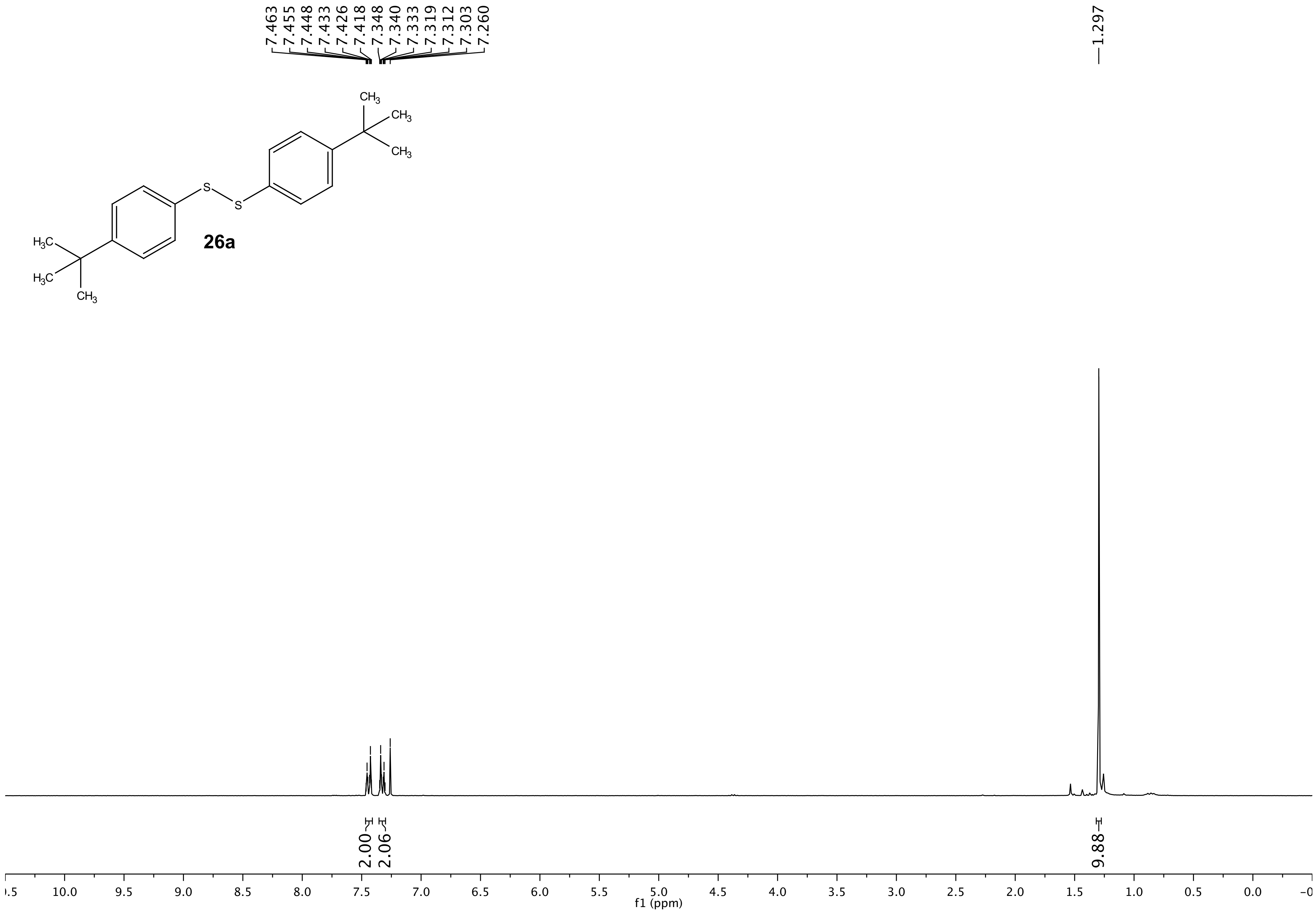
7.463
7.455
7.448
7.433
7.426
7.418
7.348
7.340
7.333
7.319
7.312
7.303
7.260
1.297
CH3
CH3
CH3
S
S
H3C
H3C
CH3
26a
2.00
2.06
9.88
10.0
9.5
9.0
8.5
8.0
7.5
7.0
6.5
6.0
5.5
5.0
4.5
4.0
3.5
3.0
2.5
2.0
1.5
1.0
0.5
0.0
f1 (ppm)

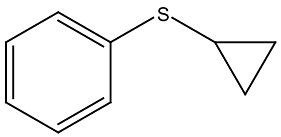

1b

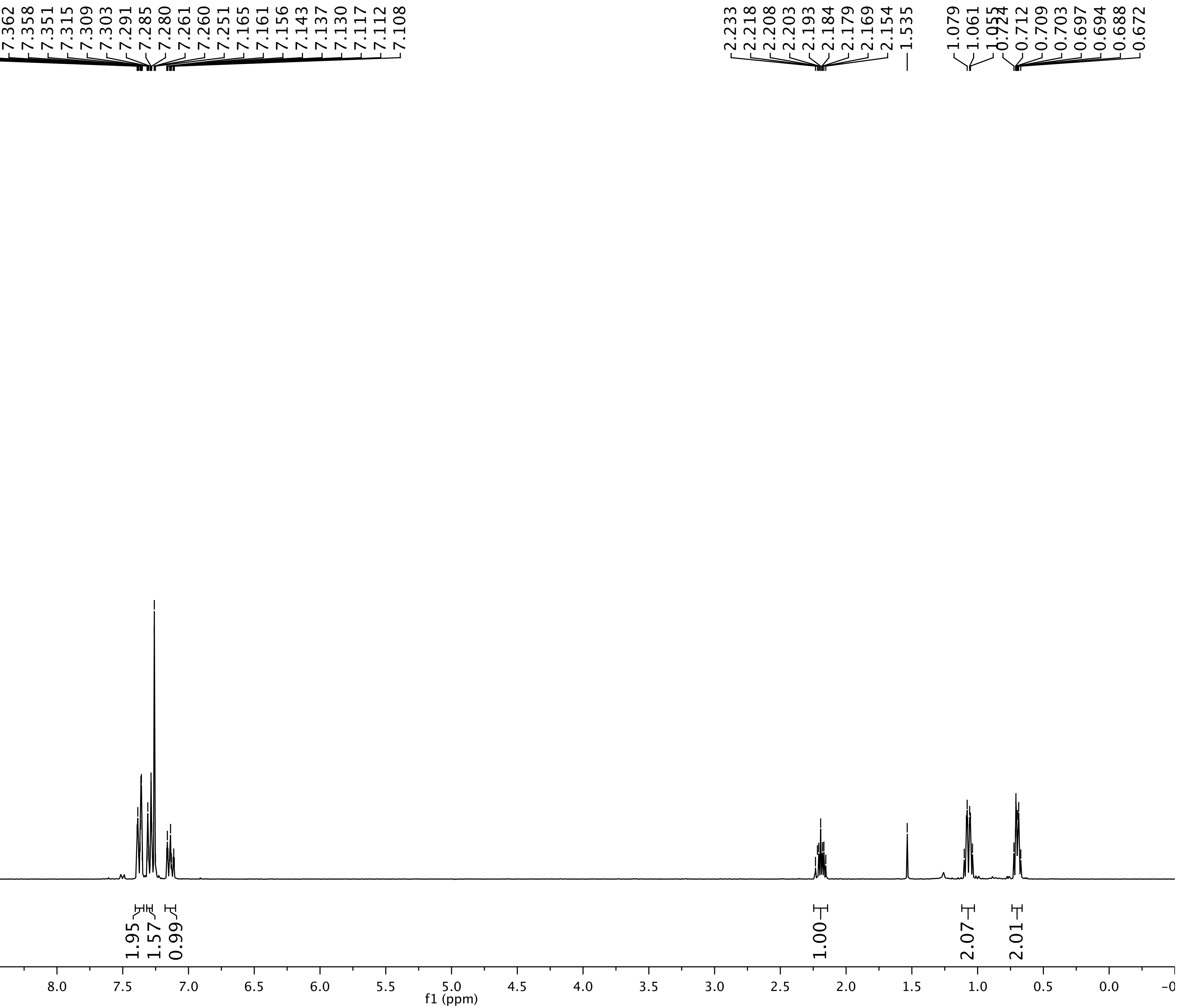
7.391
7.386
7.379
7.368
7.365
7.362
7.358
7.351
7.315
7.309
7.303
7.291
7.285
7.280
7.261
7.260
7.251
7.165
7.161
7.156
7.143
7.137
7.130
7.117
7.112
7.108
2.233
2.218
2.208
2.203
2.193
2.184
2.179
2.169
2.154
1.535
1.079
1.061
0.724
0.712
0.709
0.703
0.697
0.694
0.688
0.672
1.95
1.57
0.99
1.00
2.07
2.01
10.0
9.5
9.0
8.5
8.0
7.5
7.0
6.5
6.0
5.5
5.0
4.5
4.0
3.5
3.0
2.5
2.0
1.5
1.0
0.5
0.0
f1 (ppm)

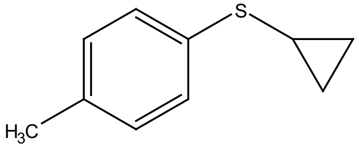

1c

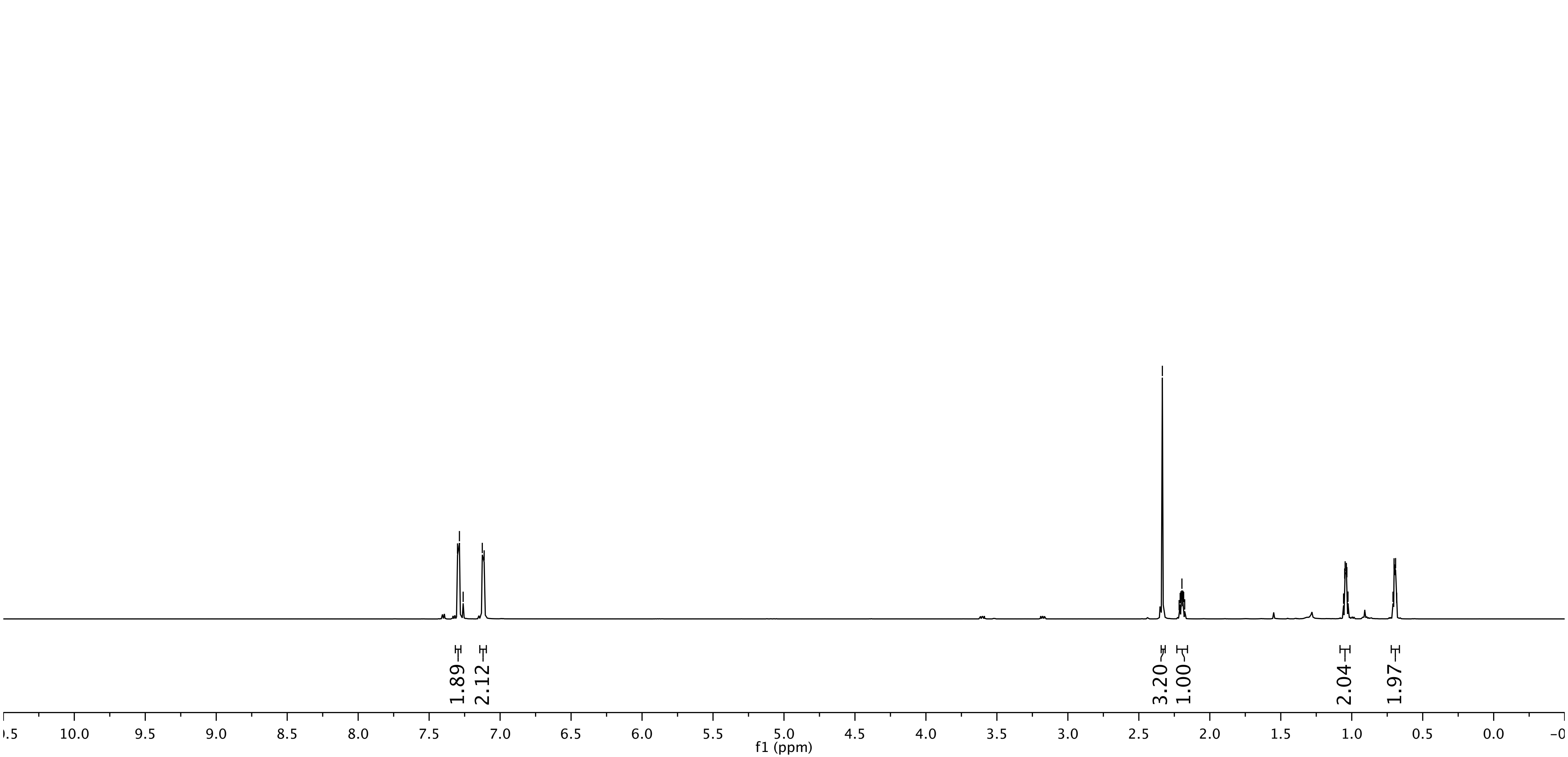
7.300
7.287
7.260
7.126
7.112
2.334
2.216
2.209
2.204
2.201
2.196
2.191
2.189
2.184
2.177
1.050
1.046
1.038
1.034
0.710
0.702
0.699
0.694
0.691
0.683
1.89
2.12
3.20
1.00
2.04
1.97
10.0
9.5
9.0
8.5
8.0
7.5
7.0
6.5
6.0
5.5
5.0
4.5
4.0
3.5
3.0
2.5
2.0
1.5
1.0
0.5
0.0
f1 (ppm)

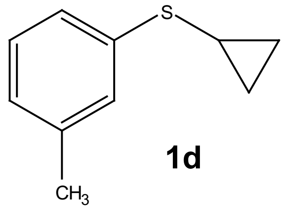
S
CH3

1d

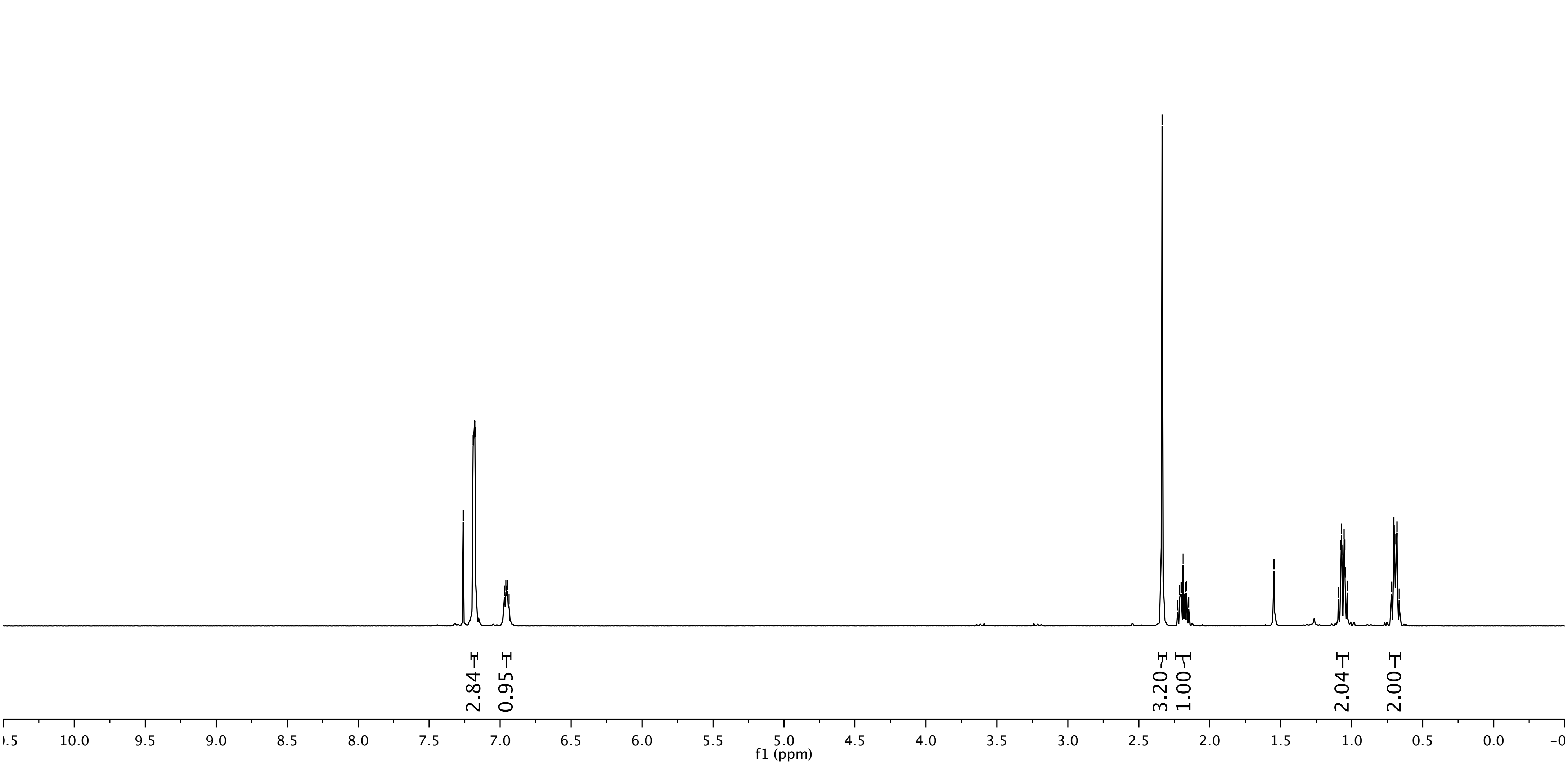
7.260
7.190
7.179
7.176
6.970
6.960
6.954
6.948
6.937
2.337
2.227
2.212
2.202
2.197
2.188
2.178
2.173
2.163
2.149
1.548
1.078
1.072
1.054
1.048
0.718
0.703
0.696
0.688
0.681
0.665
2.84
0.95
3.20
1.00
2.04
2.00
10.0
9.5
9.0
8.5
8.0
7.5
7.0
6.5
6.0
5.5
5.0
4.5
4.0
3.5
3.0
2.5
2.0
1.5
1.0
0.5
0.0
f1 (ppm)

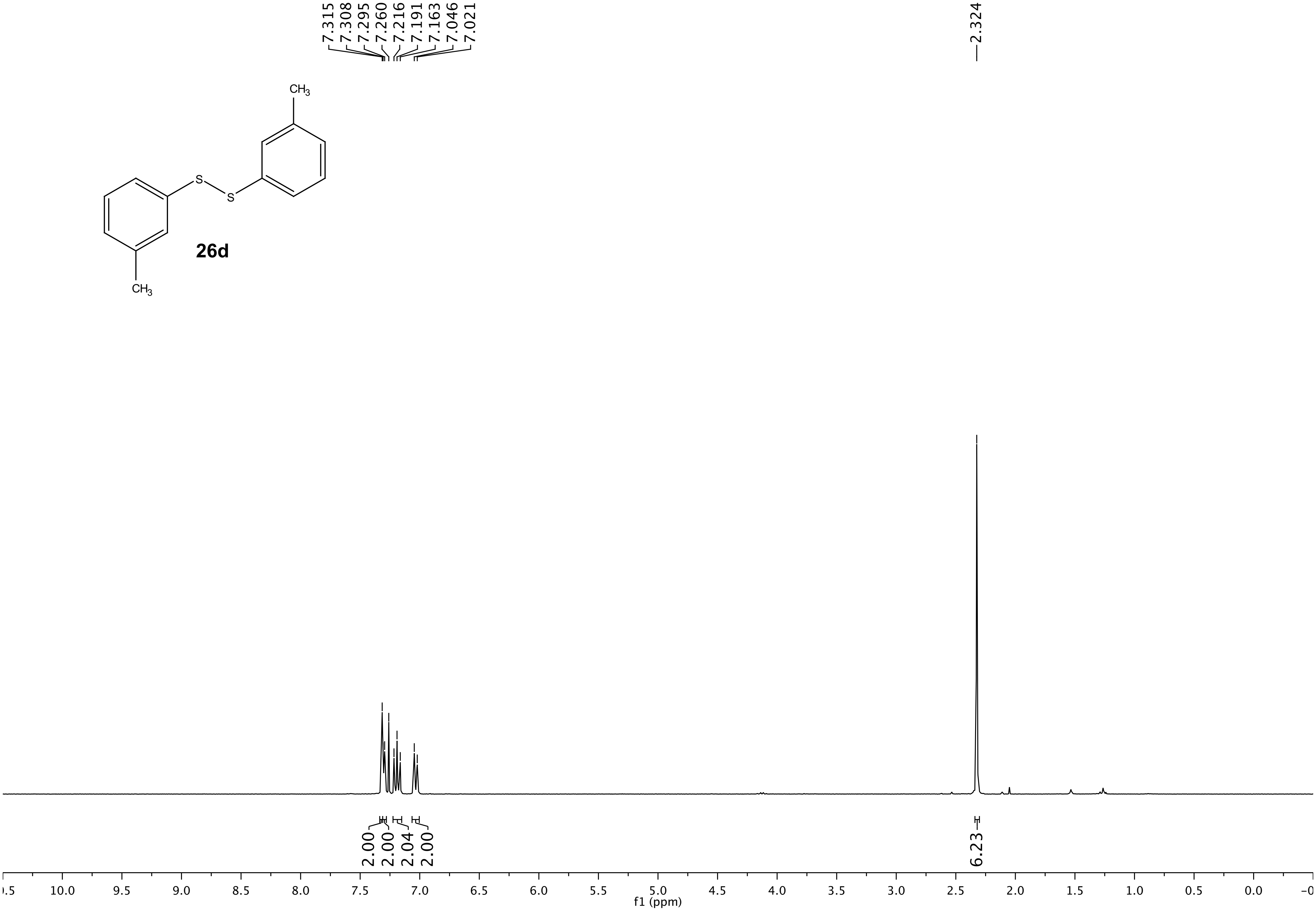

7.315
7.308
7.295
7.260
7.216
7.191
7.163
7.046
7.021
2.324
CH3
S
S
CH3
26d
2.00
2.00
2.04
2.00
6.23
10.0
9.5
9.0
8.5
8.0
7.5
7.0
6.5
6.0
5.5
5.0
4.5
4.0
3.5
3.0
2.5
2.0
1.5
1.0
0.5
0.0
f1 (ppm)

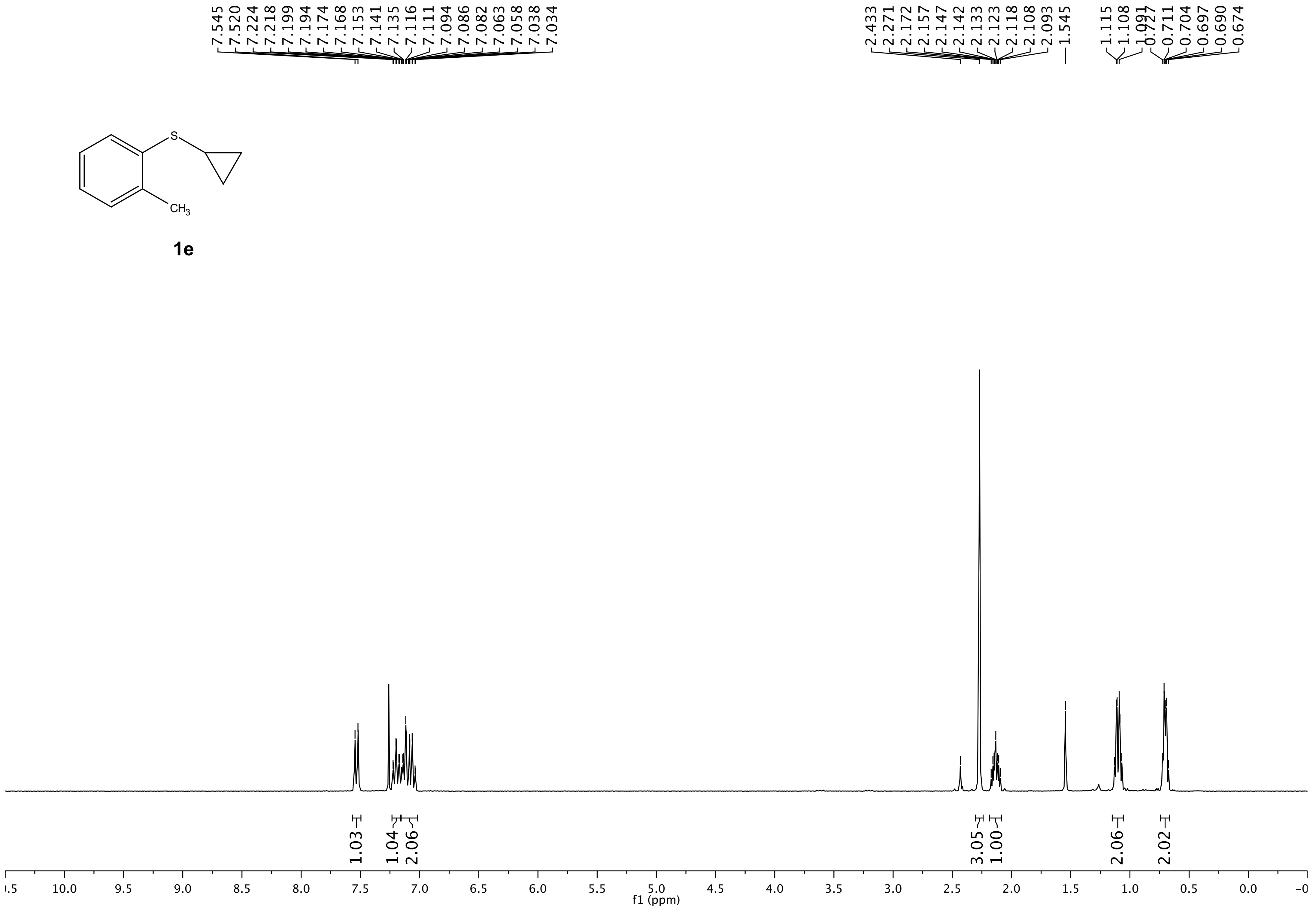
7.545
7.520
7.224
7.218
7.199
7.194
7.174
7.168
7.153
7.141
7.135
7.116
7.111
7.094
7.086
7.082
7.063
7.058
7.038
7.034
2.433
2.271
2.172
2.157
2.147
2.142
2.133
2.123
2.118
2.108
2.093
1.545
1.115
1.108
1.091
0.727
0.711
0.704
0.697
0.690
0.674
S
CH3
1.03
1.04
2.06
3.05
1.00
2.06
2.02
10.0
9.5
9.0
8.5
8.0
7.5
7.0
6.5
6.0
5.5
5.0
4.5
4.0
3.5
3.0
2.5
2.0
1.5
1.0
0.5
0.0
f1 (ppm)

1e

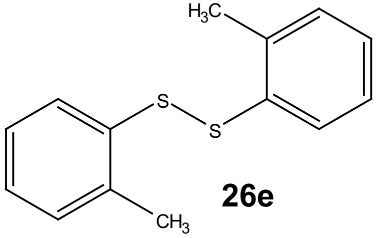

26e

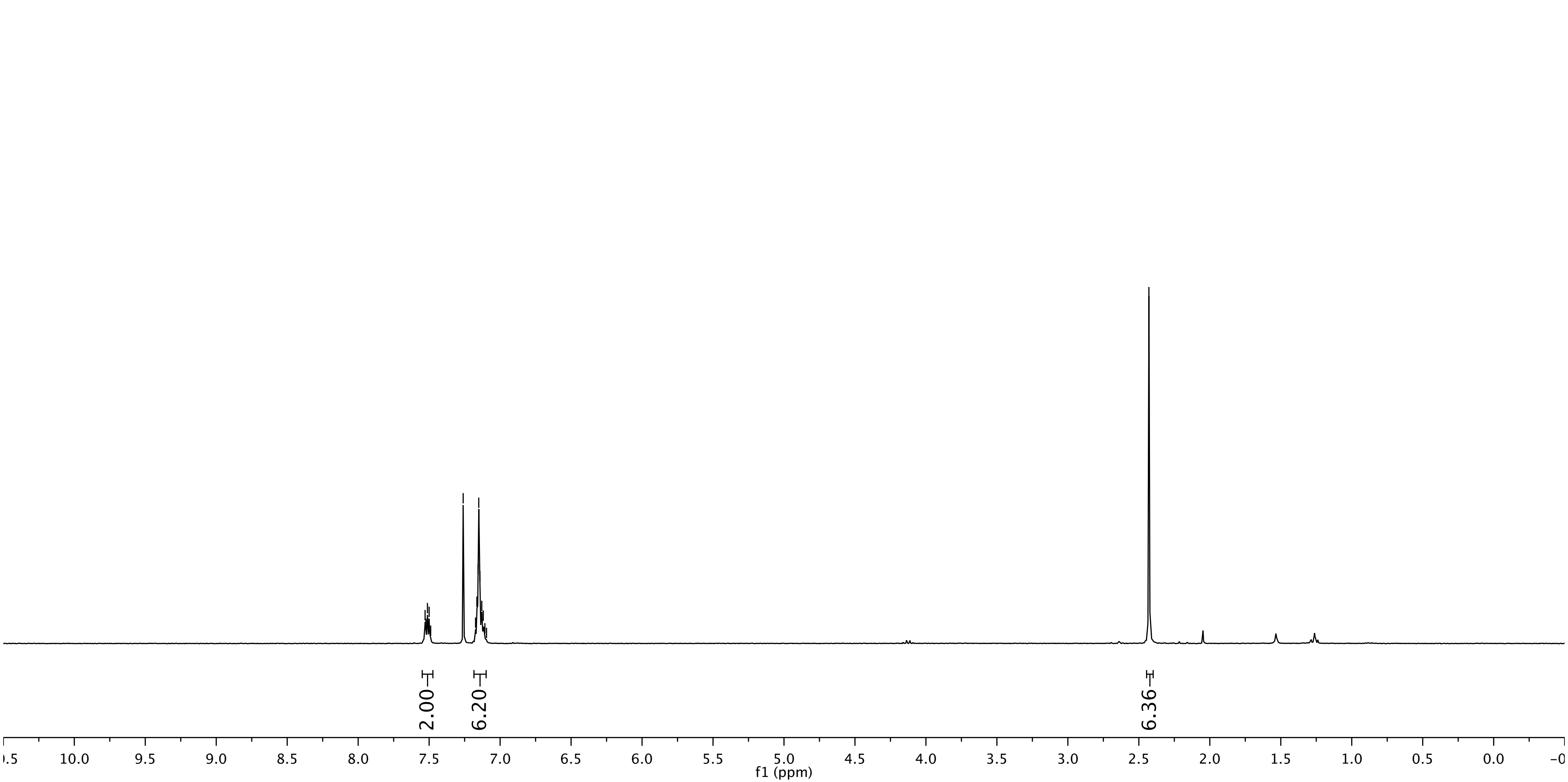

7.529
7.523
7.512
7.500
7.489
7.260
7.173
7.164
7.155
7.150
7.142
7.129
7.119
7.107
7.096
2.429
2.00
6.20
6.36
10.0
9.5
9.0
8.5
8.0
7.5
7.0
6.5
6.0
5.5
5.0
4.5
4.0
3.5
3.0
2.5
2.0
1.5
1.0
0.5
0.0
f1 (ppm)

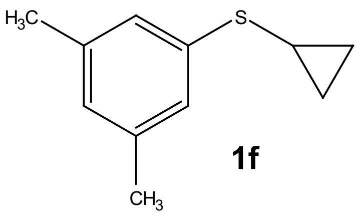
H3C
S
CH3

1f

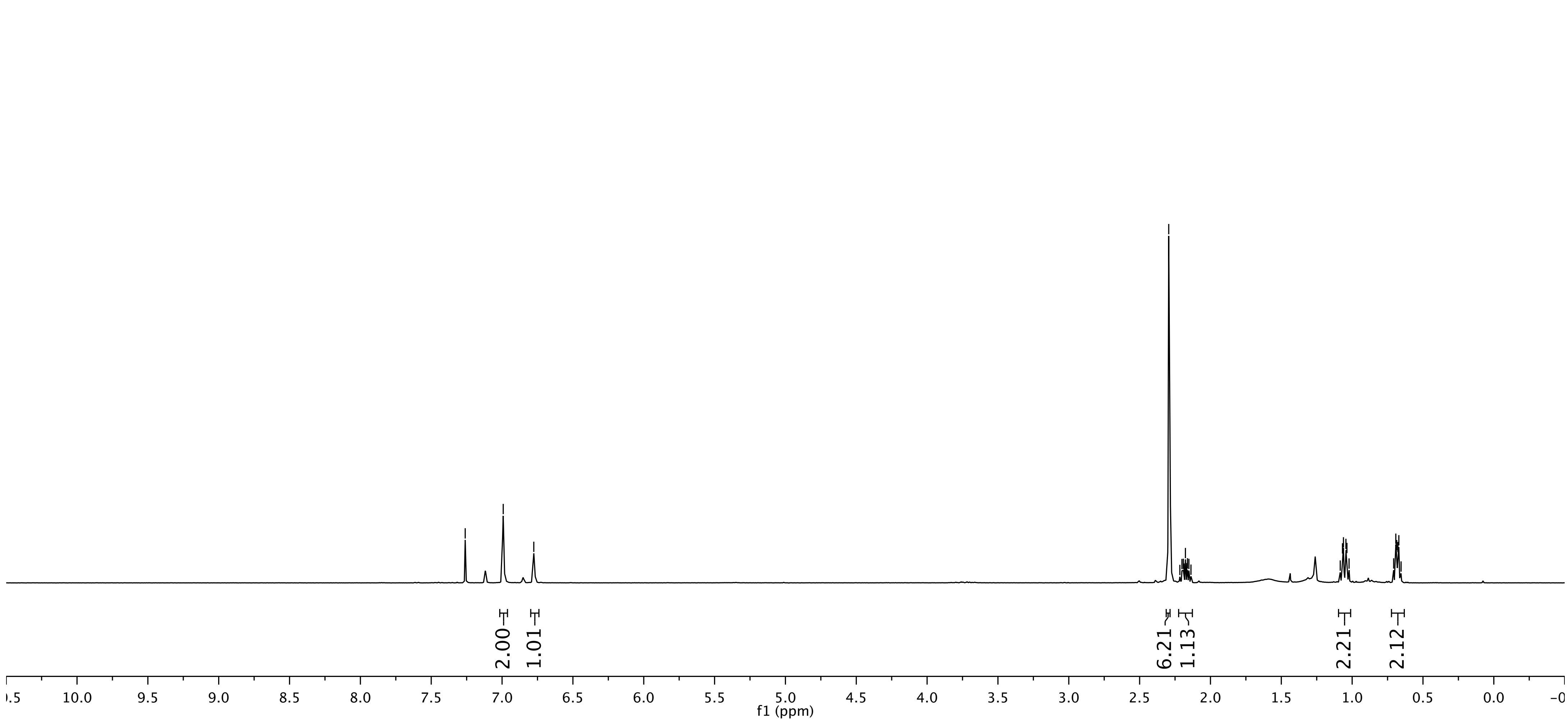
7.260
6.992
6.776
2.295
2.216
2.201
2.191
2.187
2.177
2.167
2.162
2.152
2.138
1.068
1.061
1.044
1.037
1.022
0.707
0.692
0.685
0.677
0.670
0.655
2.00
1.01
6.21
1.13
2.21
2.12
10.0
9.5
9.0
8.5
8.0
7.5
7.0
6.5
6.0
5.5
5.0
4.5
4.0
3.5
3.0
2.5
2.0
1.5
1.0
0.5
0.0
f1 (ppm)

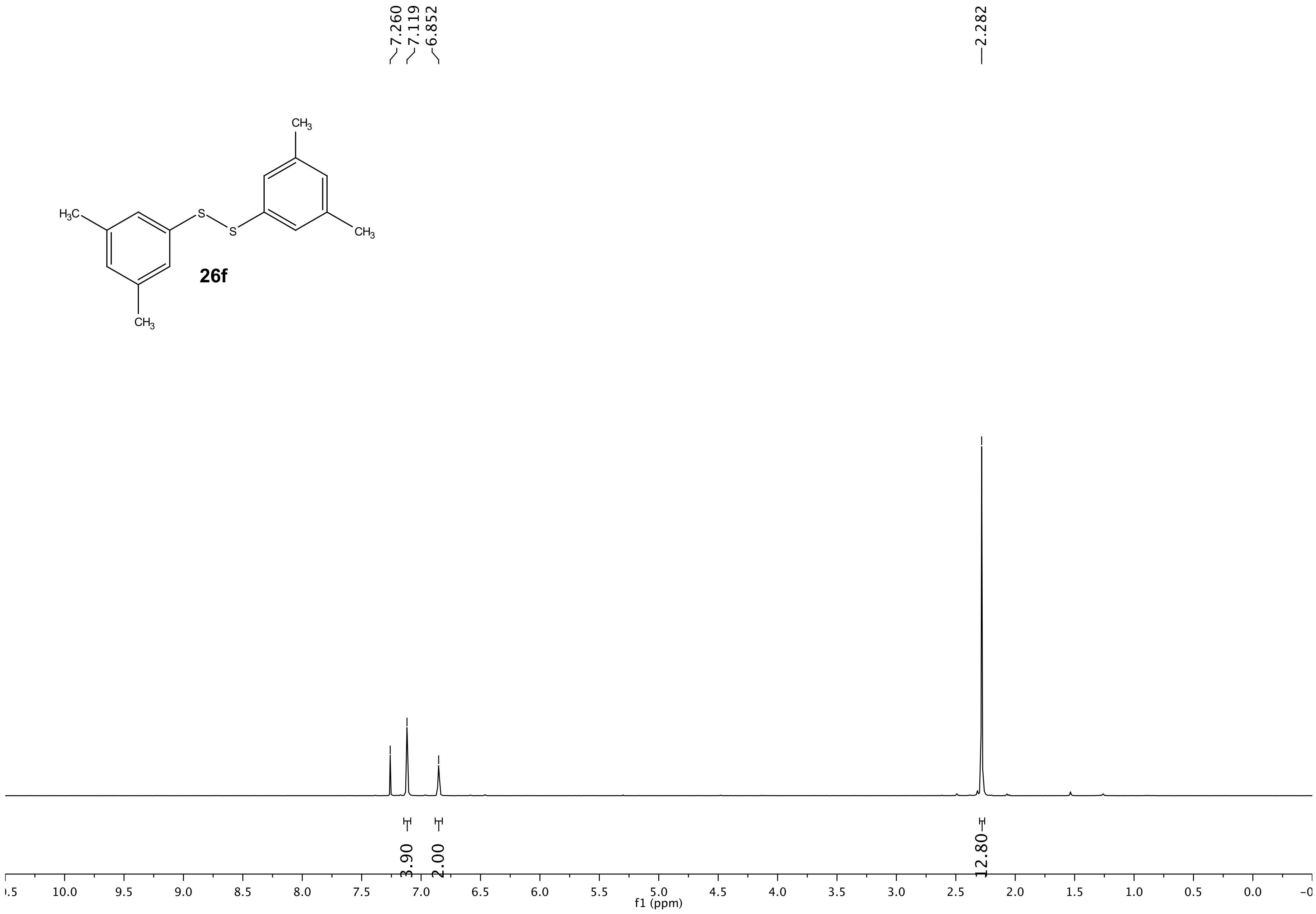

7.260
7.119
6.852
2.282
CH3
H3C
S
S
CH3
CH3
26f
3.90
2.00
12.80
10.0
9.5
9.0
8.5
8.0
7.5
7.0
6.5
6.0
5.5
5.0
4.5
4.0
3.5
3.0
2.5
2.0
1.5
1.0
0.5
0.0
f1 (ppm)

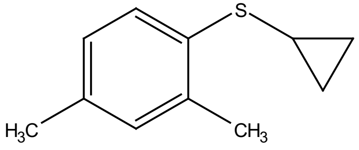
S
H_3C
CH_3

1g

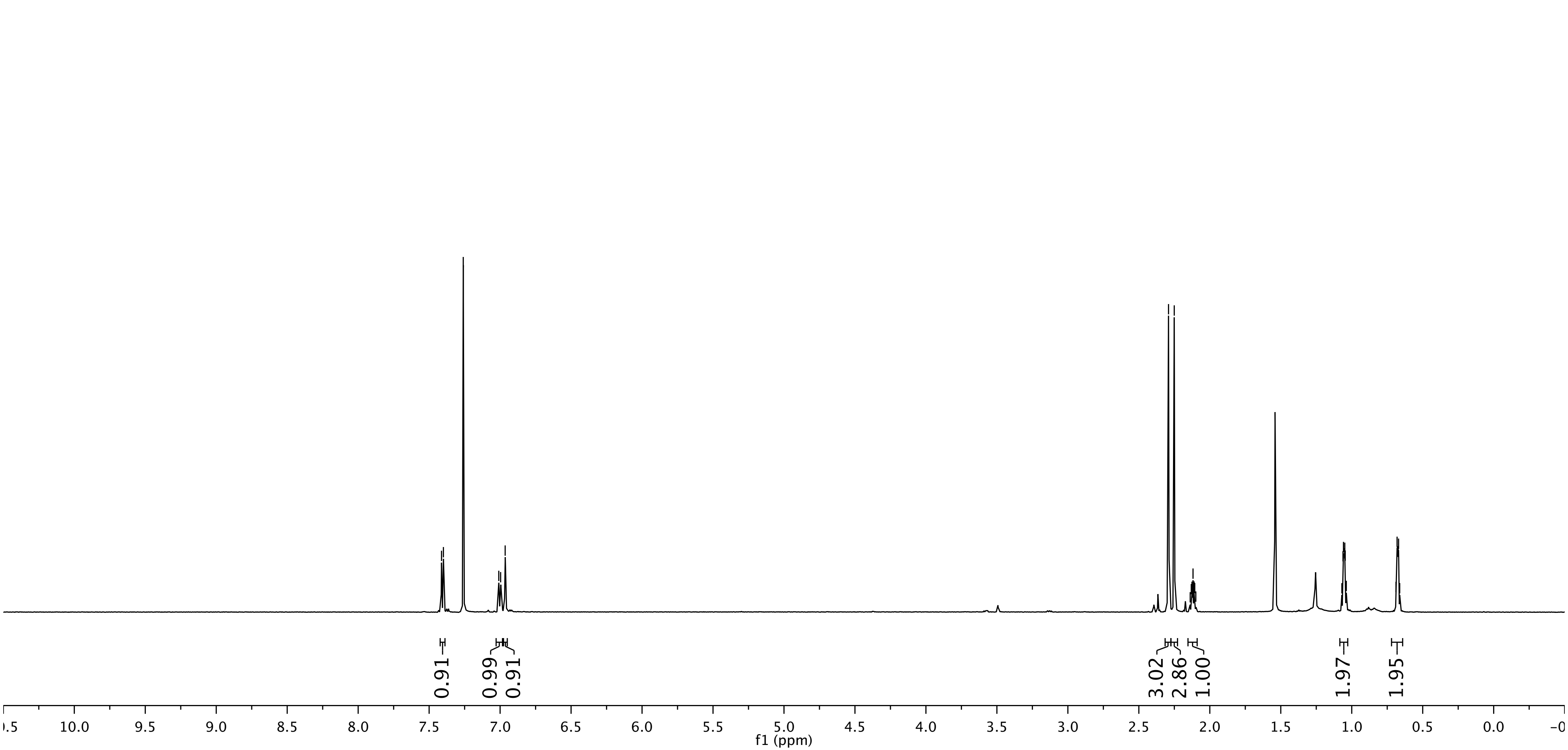
7.413
7.400
7.260
7.009
6.996
6.964
2.291
2.251
2.138
2.131
2.126
2.123
2.118
2.113
2.111
2.106
2.099
1.058
1.050
1.049
1.046
0.688
0.681
0.677
0.673
0.670
0.662
0.91
0.99
0.91
3.02
2.86
1.00
1.97
1.95
10.0 9.5 9.0 8.5 8.0 7.5 7.0 6.5 6.0 5.5 5.0 4.5 4.0 3.5 3.0 2.5 2.0 1.5 1.0 0.5 0.0
f1 (ppm)

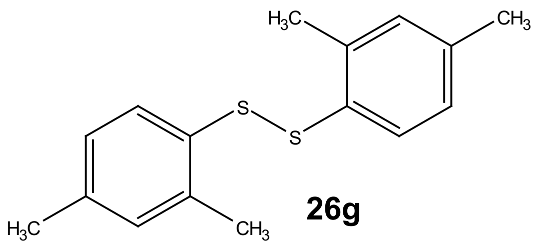

H$_3$C
CH$_3$
S
S
H$_3$C
CH$_3$

26g

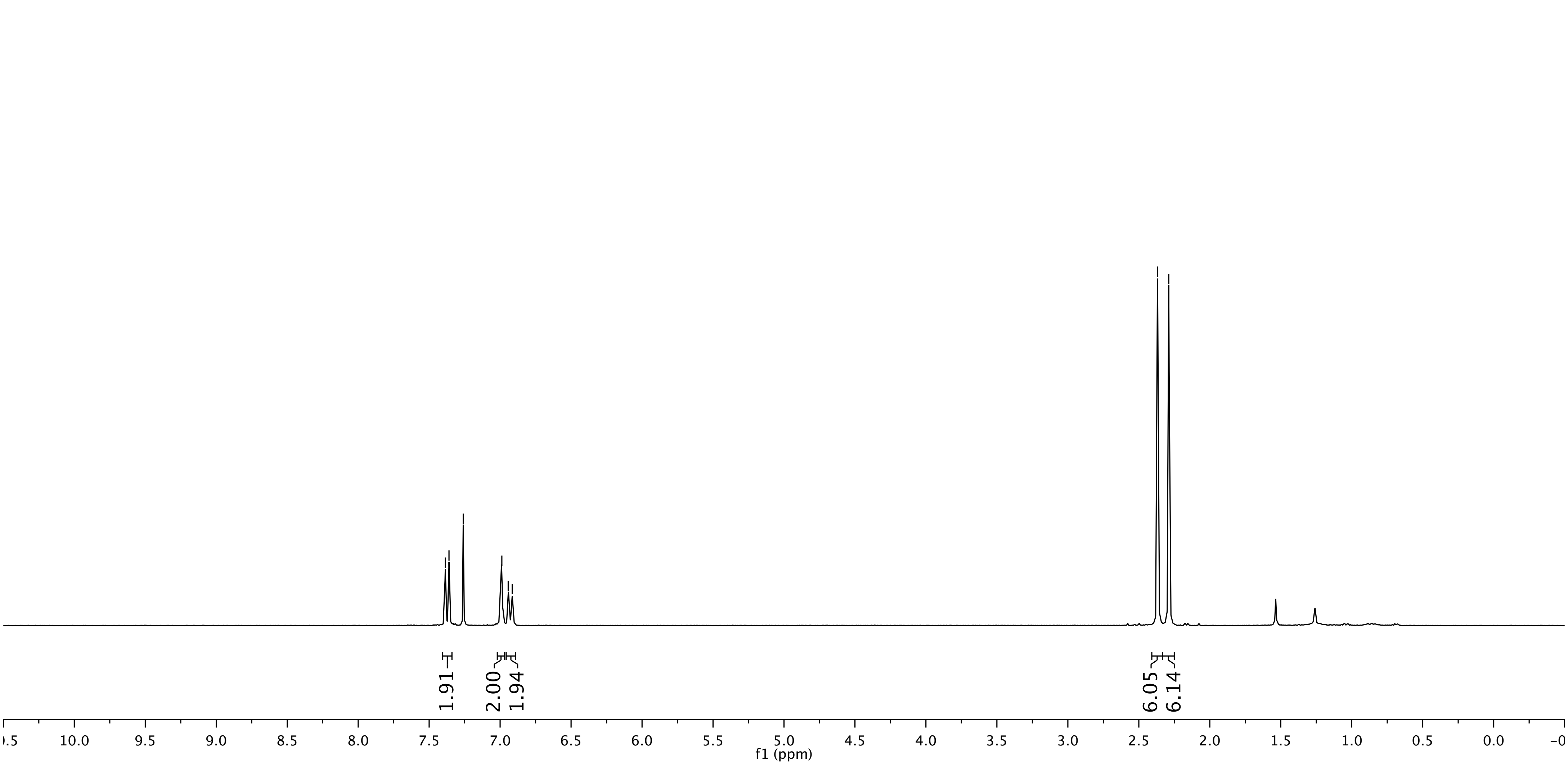

7.386
7.360
7.260
6.988
6.943
6.915
2.369
2.289
1.91
2.00
1.94
6.05
6.14
10.0
9.5
9.0
8.5
8.0
7.5
7.0
6.5
6.0
5.5
5.0
4.5
4.0
3.5
3.0
2.5
2.0
1.5
1.0
0.5
0.0
f1 (ppm)

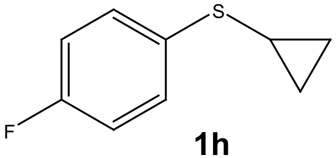

1h

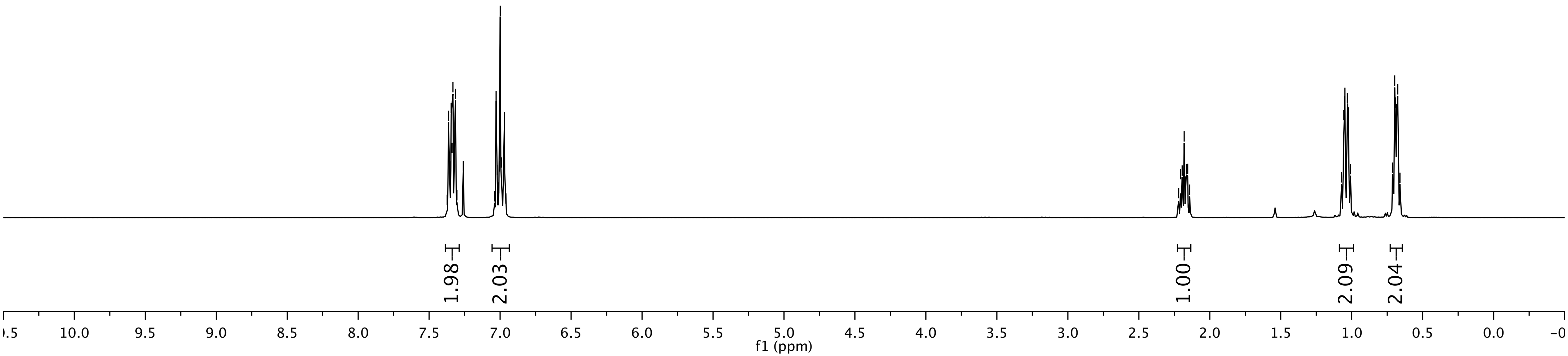
7.373
7.362
7.355
7.345
7.339
7.332
7.322
7.315
7.305
7.260
7.039
7.028
7.021
7.010
7.006
6.999
6.992
6.977
6.970
6.959
2.219
2.205
2.195
2.190
2.180
2.170
2.166
2.156
2.141
1.071
1.055
1.048
1.031
1.024
1.009
0.713
0.697
0.691
0.683
0.676
0.660
1.98
2.03
1.00
2.09
2.04
10.0
9.5
9.0
8.5
8.0
7.5
7.0
6.5
6.0
5.5
5.0
4.5
4.0
3.5
3.0
2.5
2.0
1.5
1.0
0.5
0.0
f1 (ppm)

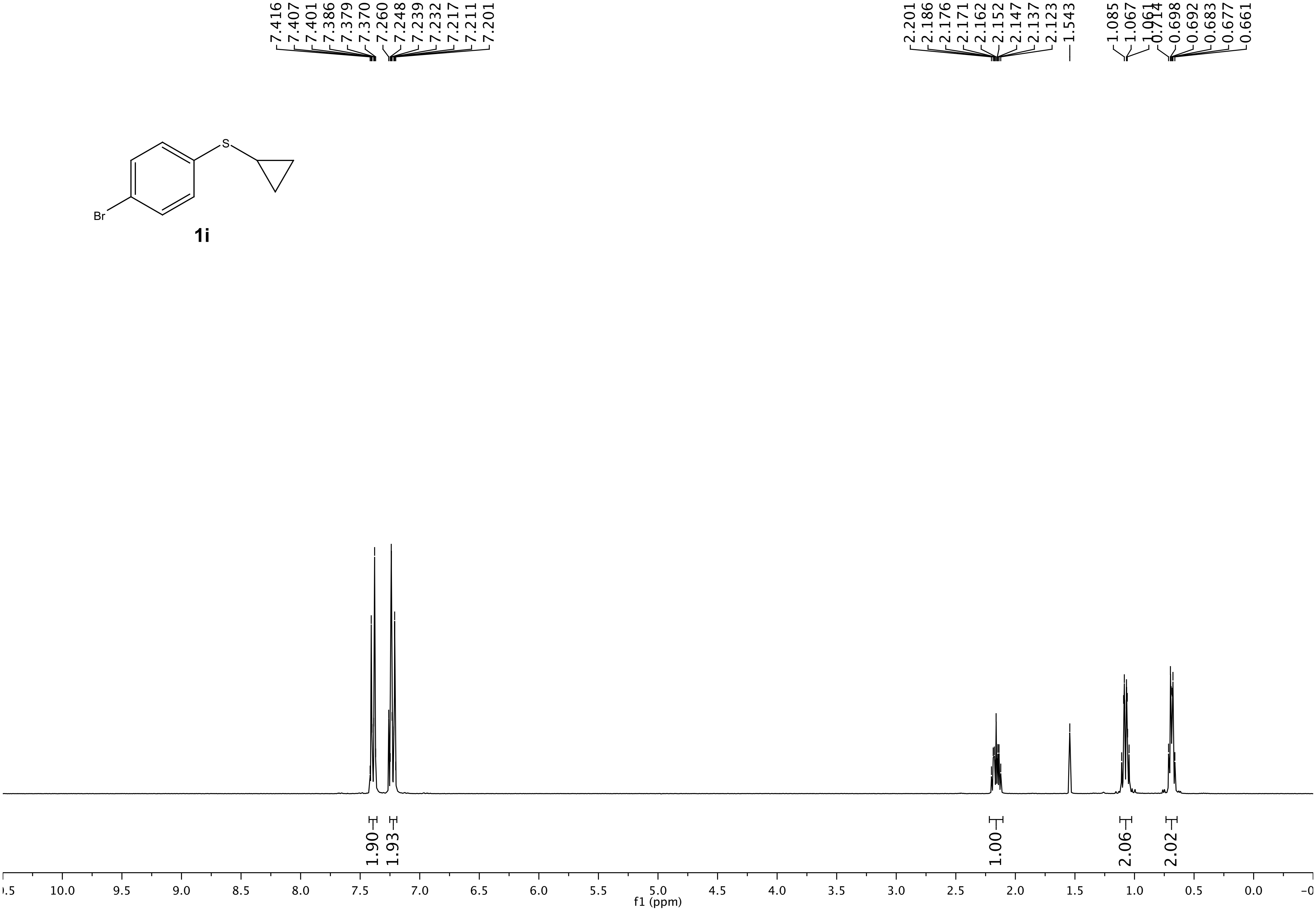
7.416
7.407
7.401
7.386
7.379
7.370
7.260
7.248
7.239
7.232
7.217
7.211
7.201
2.201
2.186
2.176
2.171
2.162
2.152
2.147
2.137
2.123
1.543
1.085
1.067
1.061
0.714
0.698
0.692
0.683
0.677
0.661
S
Br
1i
1.90
1.93
1.00
2.06
2.02
10.0
9.5
9.0
8.5
8.0
7.5
7.0
6.5
6.0
5.5
5.0
4.5
4.0
3.5
3.0
2.5
2.0
1.5
1.0
0.5
0.0
f1 (ppm)

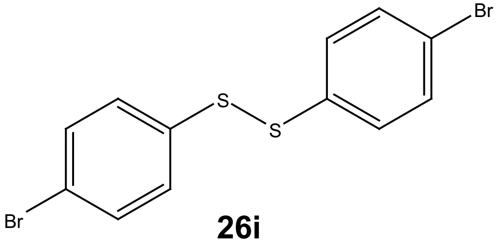

26i

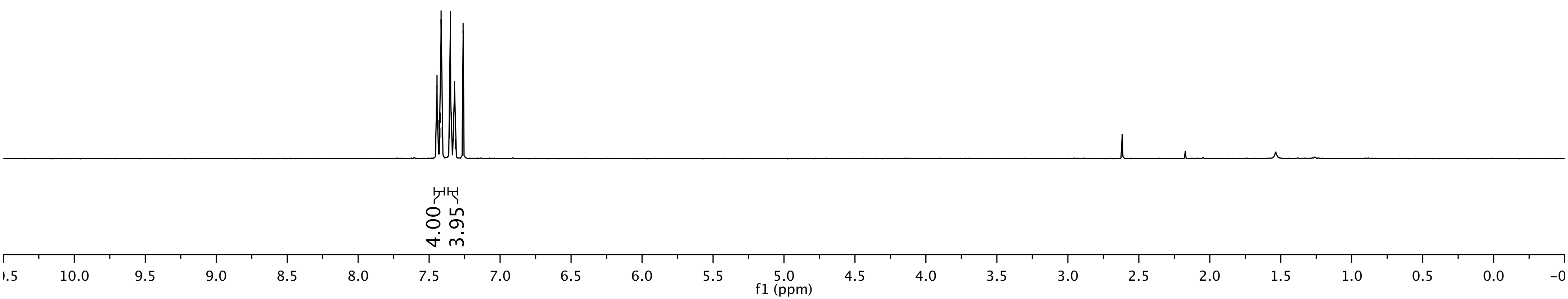
7.453
7.444
7.438
7.424
7.423
7.416
7.408
7.358
7.350
7.343
7.328
7.322
7.313
7.261
4.00
3.95
10.0 9.5 9.0 8.5 8.0 7.5 7.0 6.5 6.0 5.5 5.0 4.5 4.0 3.5 3.0 2.5 2.0 1.5 1.0 0.5 0.0
f1 (ppm)

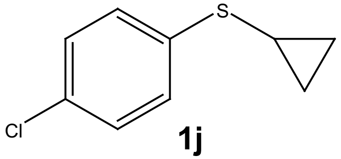
S
Cl

1j

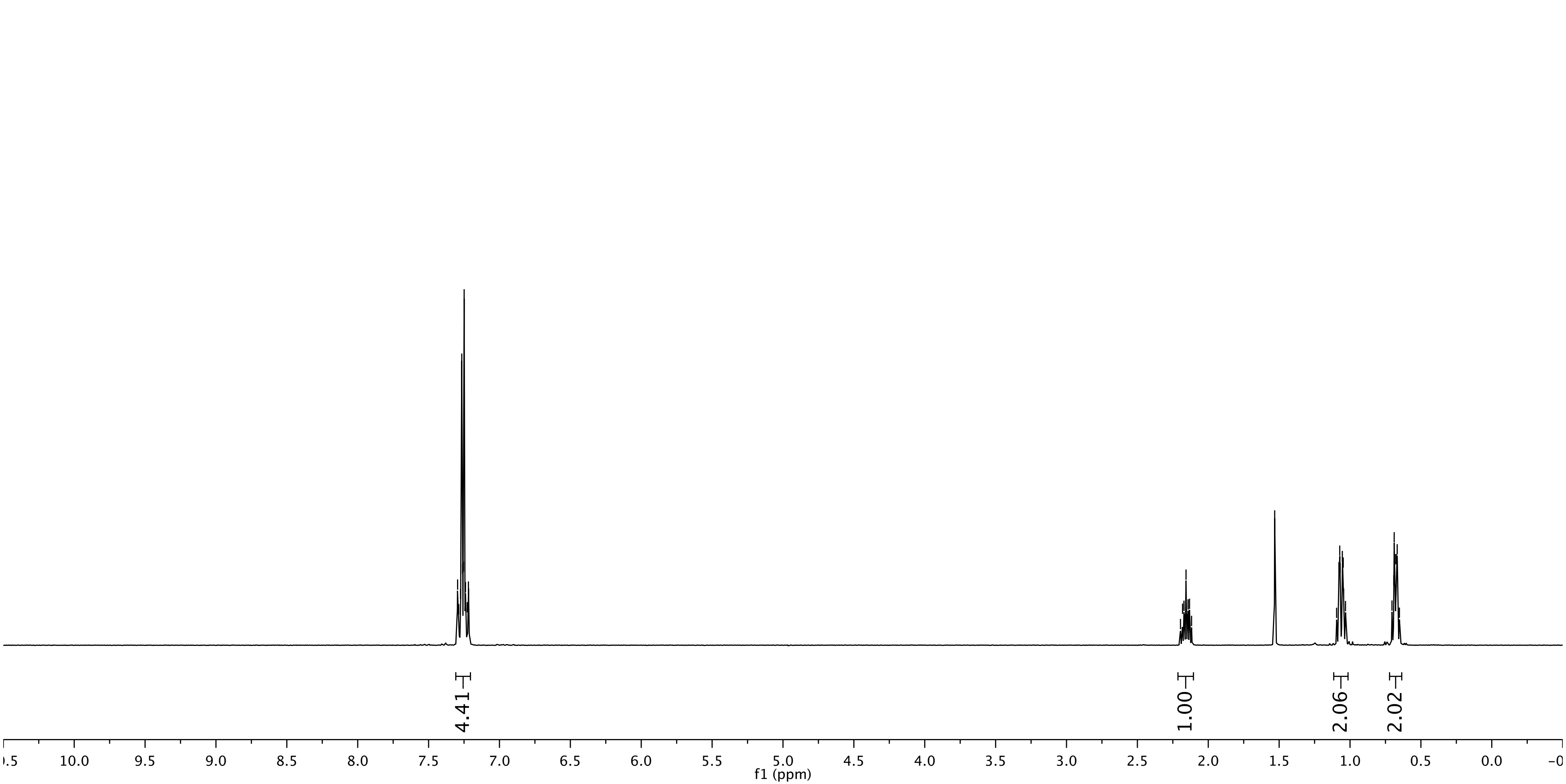
7.295
7.287
7.274
7.266
7.260
7.254
7.249
7.240
7.227
7.219
2.196
2.181
2.171
2.167
2.157
2.147
2.142
2.132
2.118
1.531
1.073
1.054
0.704
0.691
0.688
0.682
0.677
0.674
0.667
0.652
4.41
1.00
2.06
2.02
10.0
9.5
9.0
8.5
8.0
7.5
7.0
6.5
6.0
5.5
5.0
4.5
4.0
3.5
3.0
2.5
2.0
1.5
1.0
0.5
0.0
f1 (ppm)

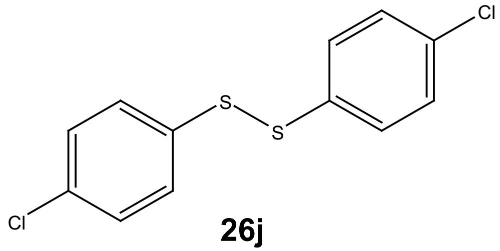
Cl
S
S
Cl

26j

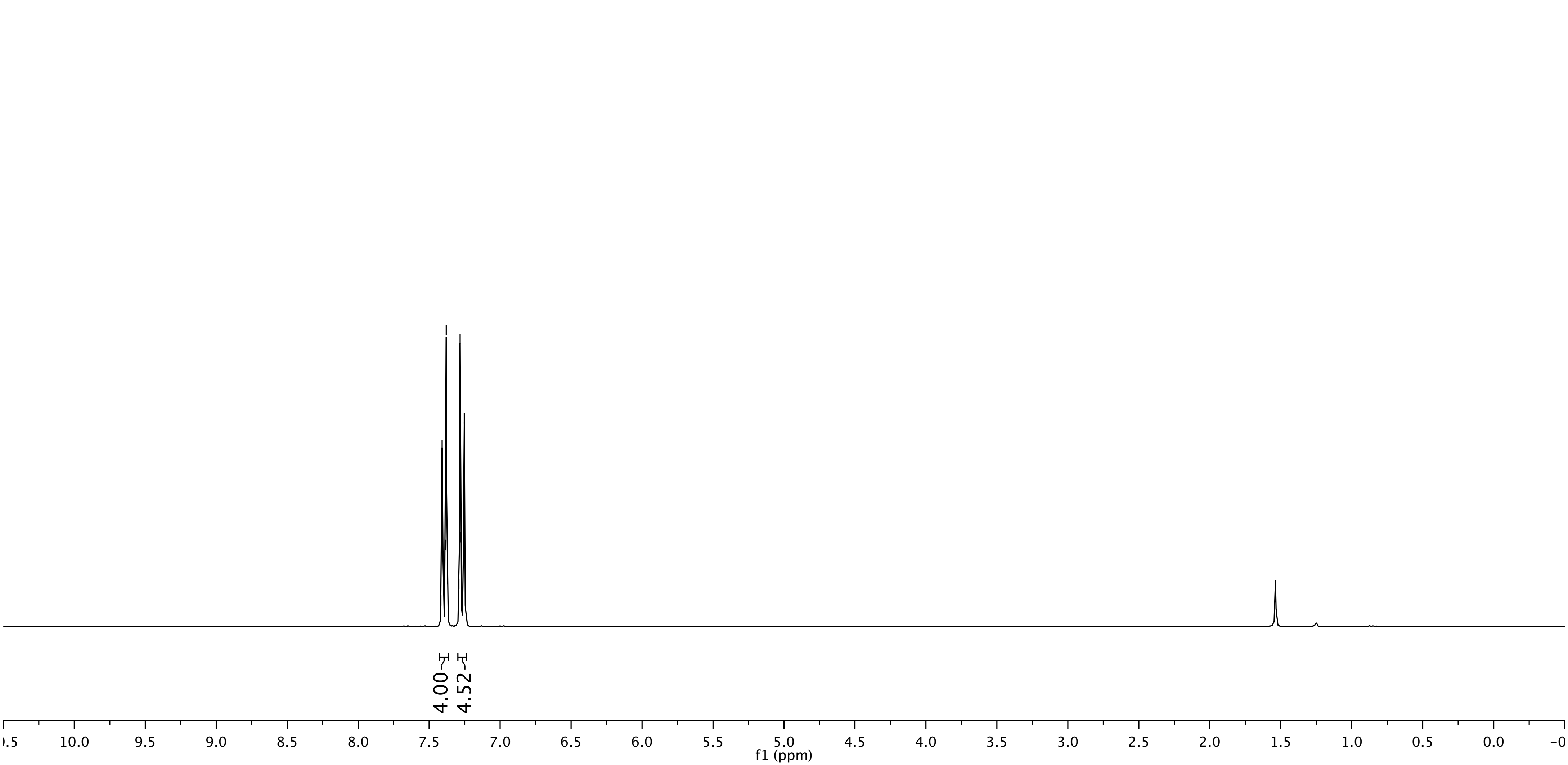
7.417
7.408
7.402
7.387
7.380
7.371
7.290
7.282
7.275
7.260
7.253
7.244
4.00
4.52
10.0
9.5
9.0
8.5
8.0
7.5
7.0
6.5
6.0
5.5
5.0
4.5
4.0
3.5
3.0
2.5
2.0
1.5
1.0
0.5
0.0
f1 (ppm)

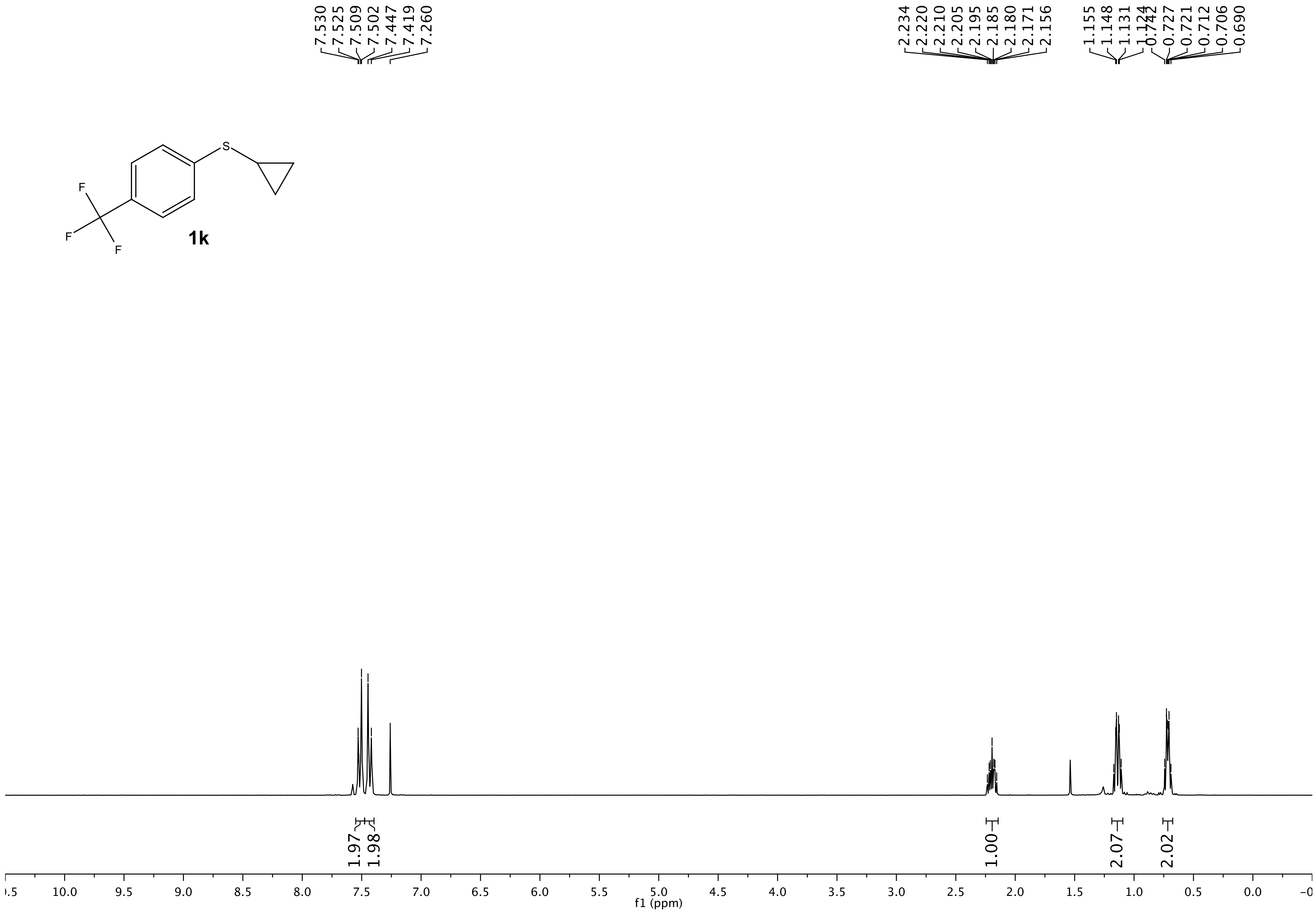
7.530
7.525
7.509
7.502
7.447
7.419
7.260
2.234
2.220
2.210
2.205
2.195
2.185
2.180
2.171
2.156
1.155
1.148
1.131
1.124
0.742
0.727
0.721
0.712
0.706
0.690
1k
1.97
1.98
1.00
2.07
2.02
10.0
9.5
9.0
8.5
8.0
7.5
7.0
6.5
6.0
5.5
5.0
4.5
4.0
3.5
3.0
2.5
2.0
1.5
1.0
0.5
0.0
f1 (ppm)

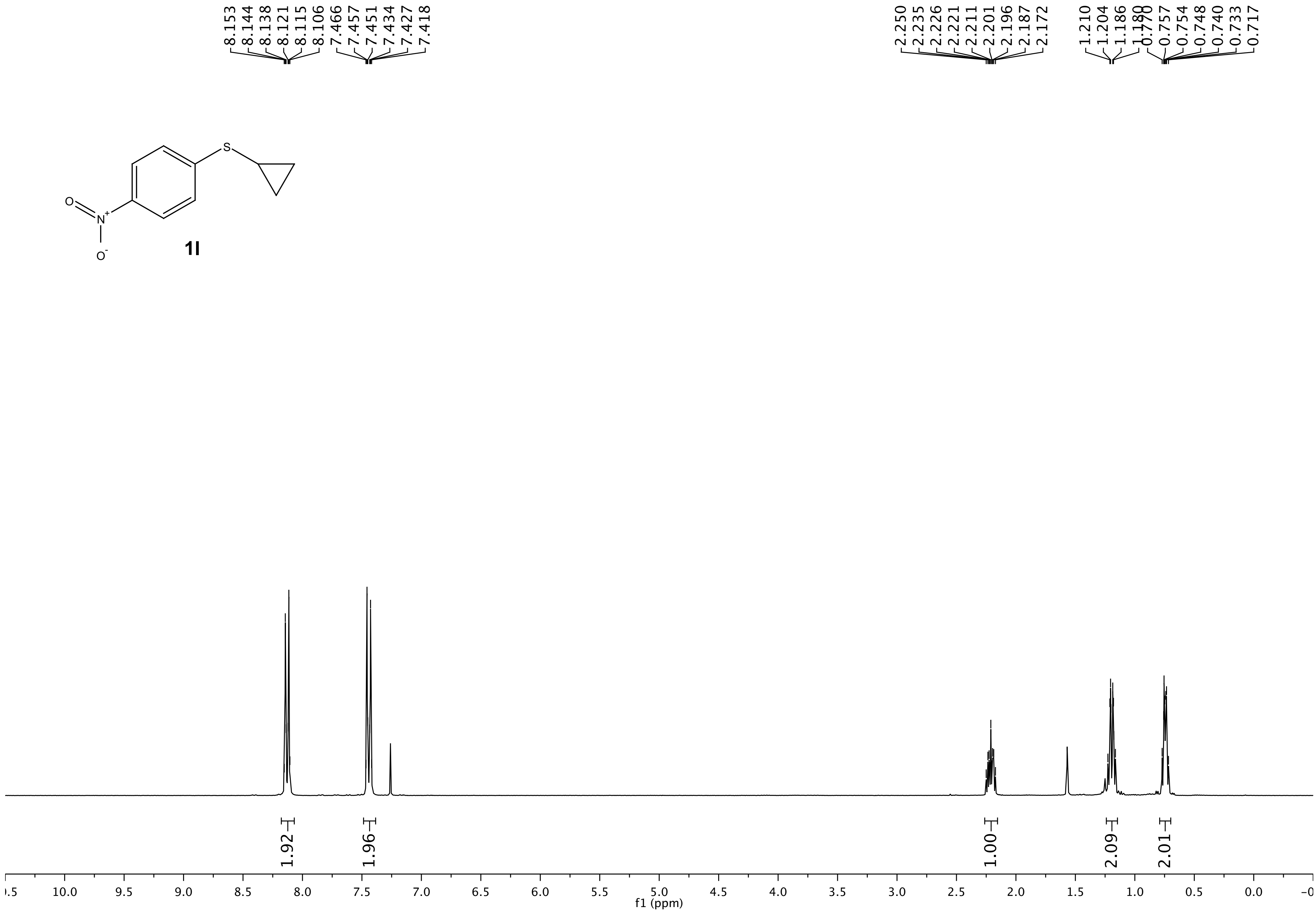
8.153
8.144
8.138
8.121
8.115
8.106
7.466
7.457
7.451
7.434
7.427
7.418
2.250
2.235
2.226
2.221
2.211
2.201
2.196
2.187
2.172
1.210
1.204
1.186
1.180
0.770
0.757
0.754
0.748
0.740
0.733
0.717
S
O
N+
O-
1l
1.92
1.96
1.00
2.09
2.01
10.0
9.5
9.0
8.5
8.0
7.5
7.0
6.5
6.0
5.5
5.0
4.5
4.0
3.5
3.0
2.5
2.0
1.5
1.0
0.5
0.0
f1 (ppm)

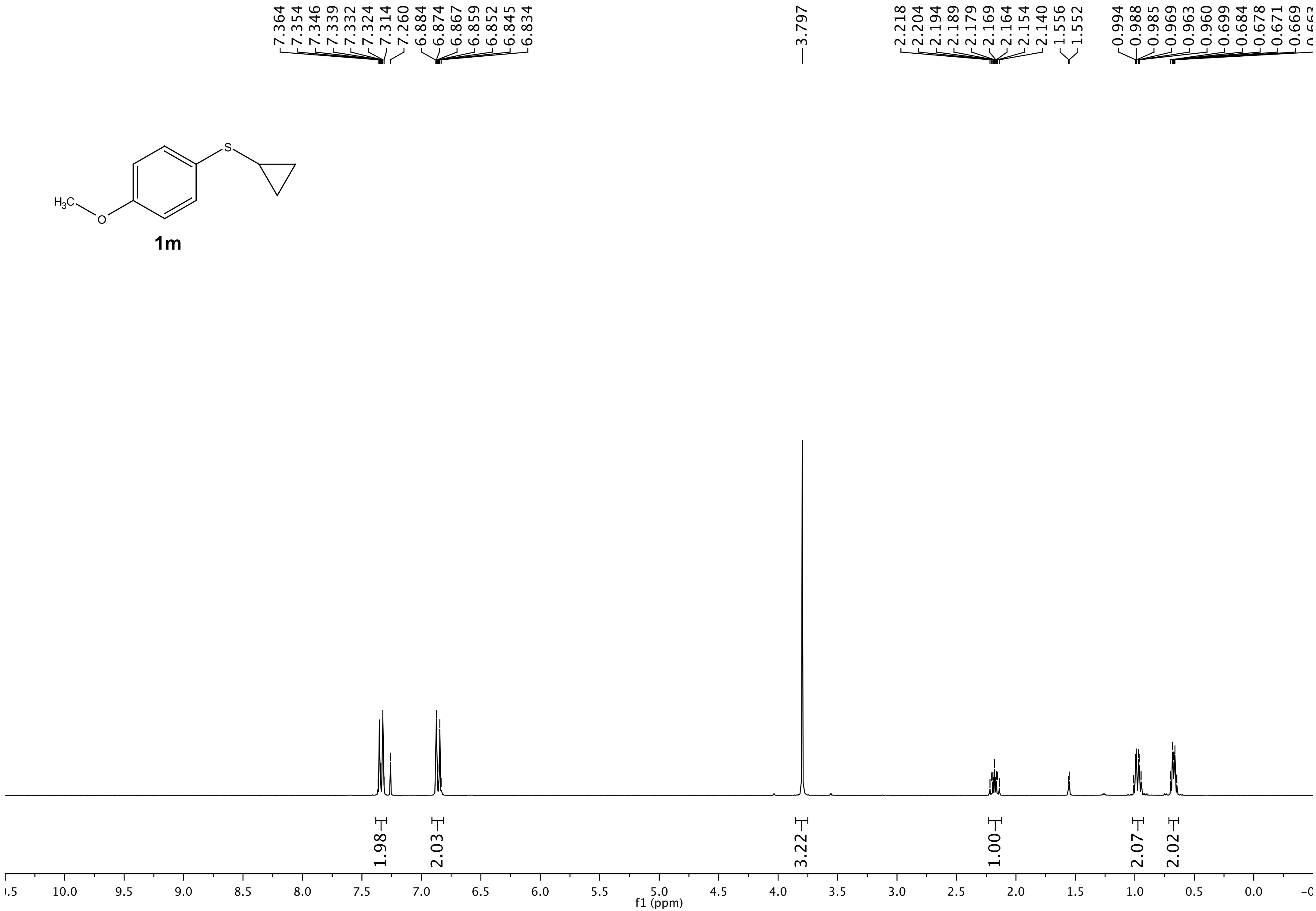
7.364
7.354
7.346
7.339
7.332
7.324
7.314
7.260
6.884
6.874
6.867
6.859
6.852
6.845
6.834
3.797
2.218
2.204
2.194
2.189
2.179
2.169
2.164
2.154
2.140
1.556
1.552
0.994
0.988
0.985
0.969
0.963
0.960
0.699
0.684
0.678
0.671
0.669
S
H_3C
O
1m
1.98
2.03
3.22
1.00
2.07
2.02
10.0
9.5
9.0
8.5
8.0
7.5
7.0
6.5
6.0
5.5
5.0
4.5
4.0
3.5
3.0
2.5
2.0
1.5
1.0
0.5
0.0
f1 (ppm)

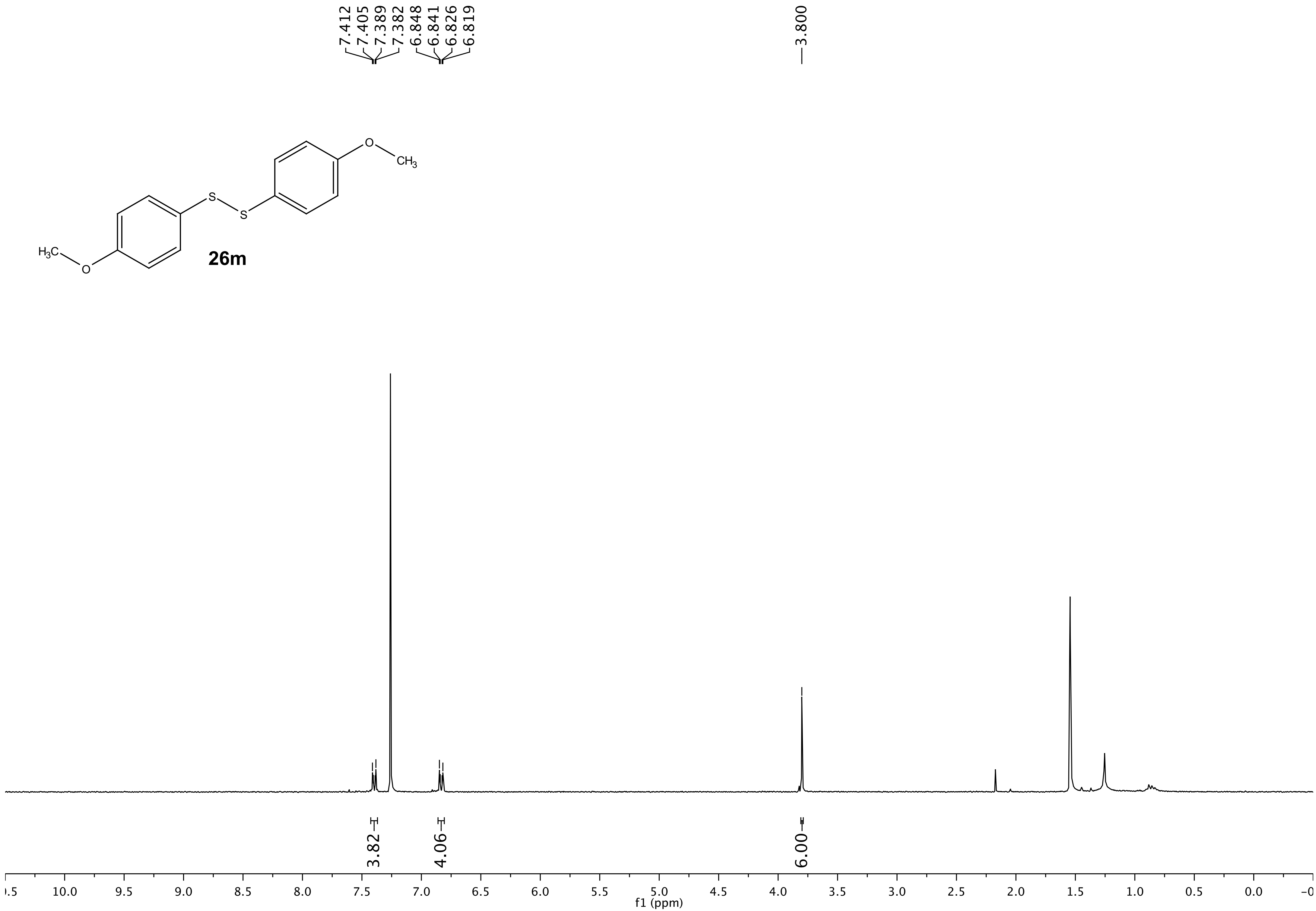
7.412
7.405
7.389
7.382
6.848
6.841
6.826
6.819
3.800
H3C
O
S
S
O
CH3
26m
3.82
4.06
6.00
f1 (ppm)

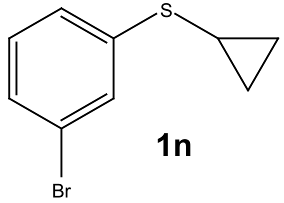
S
Br

1n

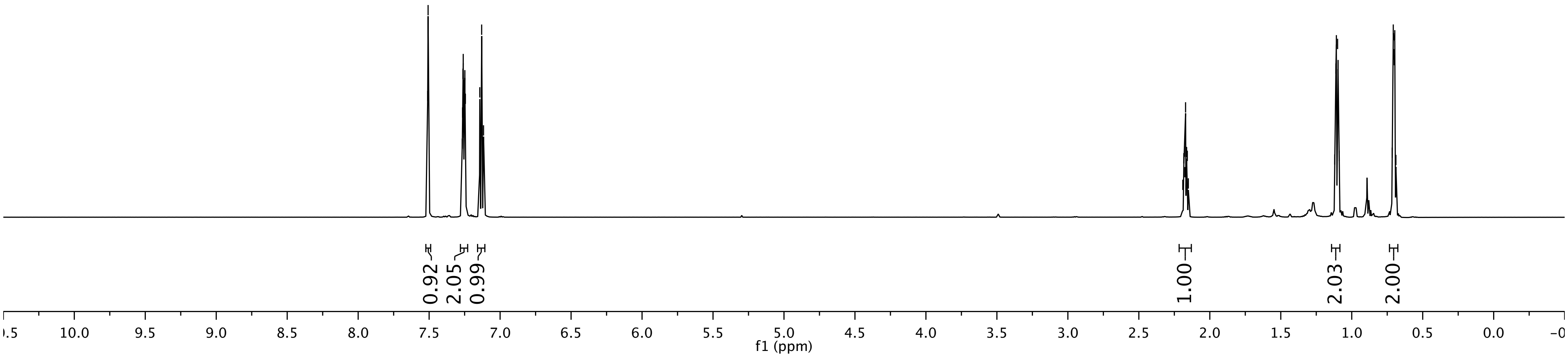
7.510
7.507
7.504
7.267
7.265
7.262
7.260
7.258
7.257
7.256
7.254
7.252
7.248
7.245
7.143
7.130
7.117
2.190
2.183
2.178
2.176
2.171
2.166
2.163
2.159
2.151
1.112
1.108
1.100
1.096
0.715
0.707
0.704
0.700
0.697
0.689
0.92
2.05
0.99
1.00
2.03
2.00
10.0
9.5
9.0
8.5
8.0
7.5
7.0
6.5
6.0
5.5
5.0
4.5
4.0
3.5
3.0
2.5
2.0
1.5
1.0
0.5
0.0
f1 (ppm)

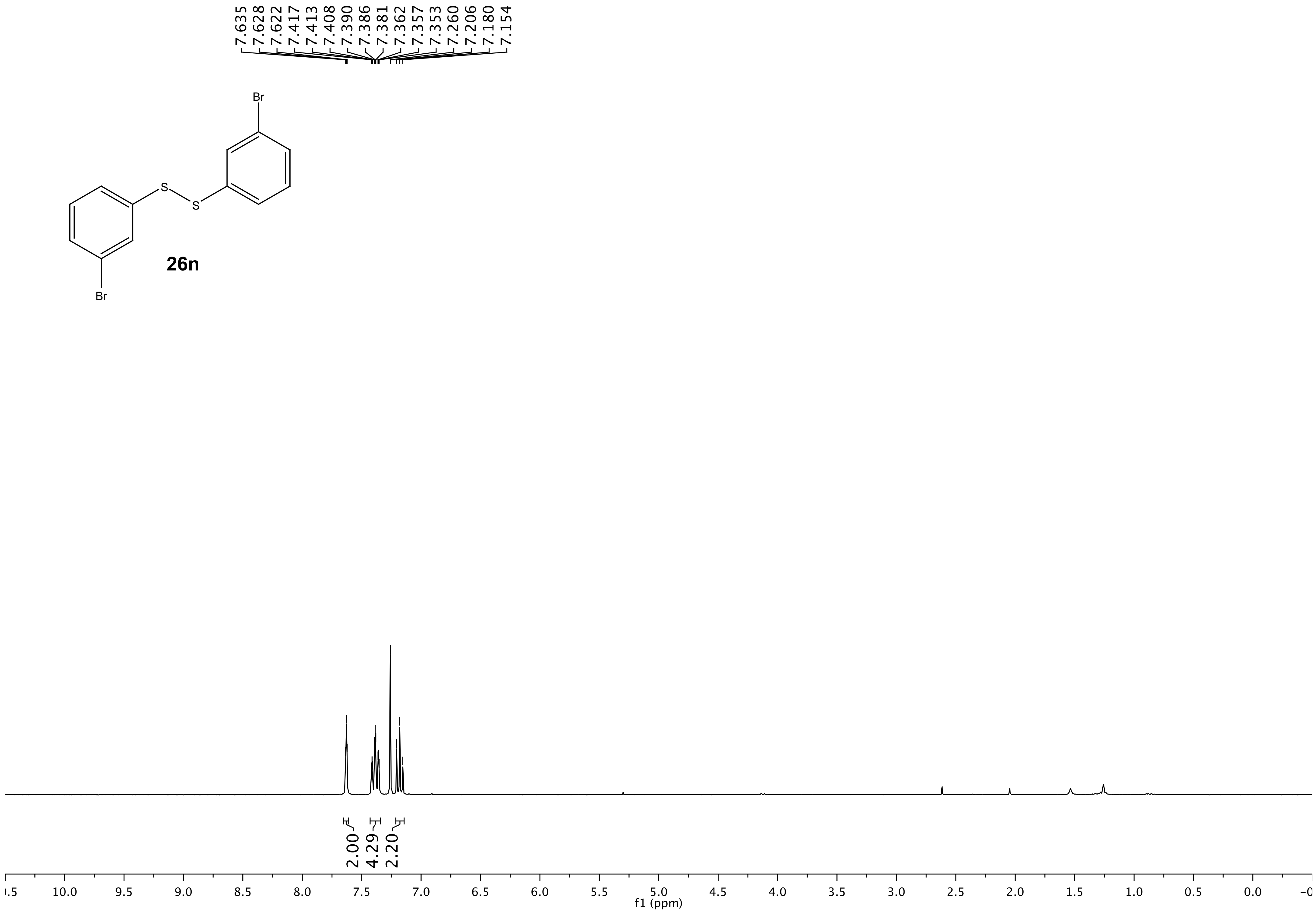
7.635
7.628
7.622
7.417
7.413
7.408
7.390
7.386
7.381
7.362
7.357
7.353
7.260
7.206
7.180
7.154
Br
S
S
Br
26n
2.00
4.29
2.20
10.0
9.5
9.0
8.5
8.0
7.5
7.0
6.5
6.0
5.5
5.0
4.5
4.0
3.5
3.0
2.5
2.0
1.5
1.0
0.5
0.0
f1 (ppm)

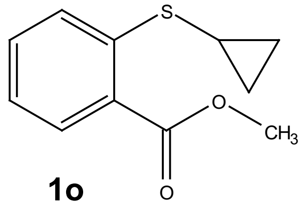
S
O
CH3
O

1o

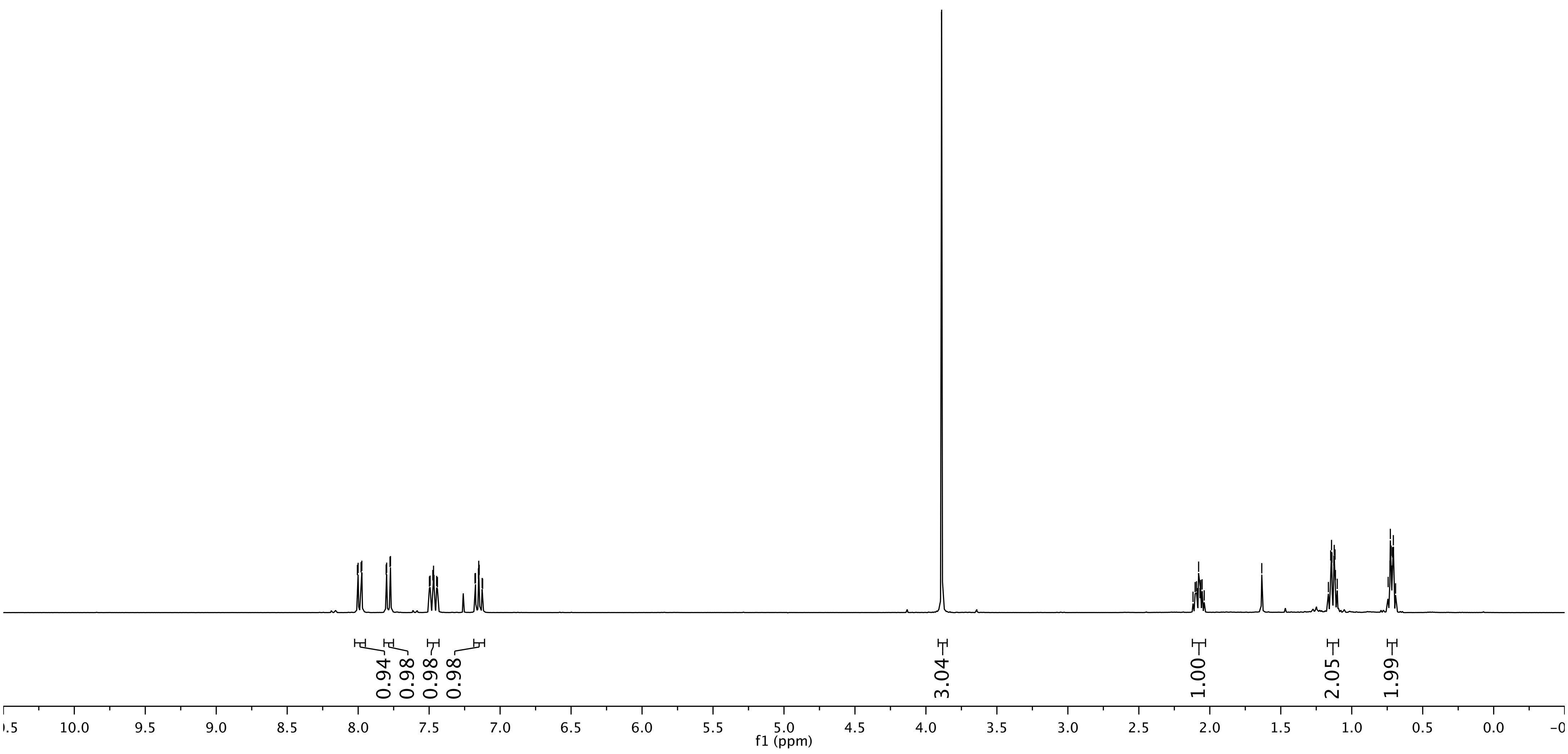
8.006
8.000
7.979
7.974
7.803
7.799
7.776
7.772
7.498
7.493
7.474
7.471
7.469
7.466
7.447
7.442
7.177
7.173
7.153
7.151
7.147
7.127
7.123
3.889
2.118
2.103
2.094
2.088
2.079
2.069
2.064
2.054
2.039
1.634
1.148
1.142
1.123
1.118
0.744
0.731
0.728
0.722
0.716
0.713
0.707
0.691
0.94
0.98
0.98
0.98
3.04
1.00
2.05
1.99
10.0
9.5
9.0
8.5
8.0
7.5
7.0
6.5
6.0
5.5
5.0
4.5
4.0
3.5
3.0
2.5
2.0
1.5
1.0
0.5
0.0
f1 (ppm)

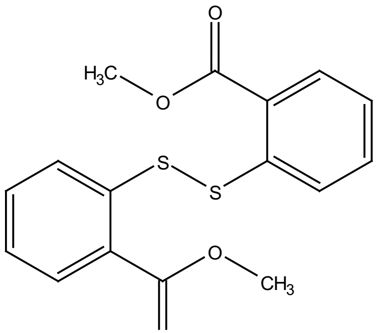
H3C
O
O
S
S
O
CH3
O

26o

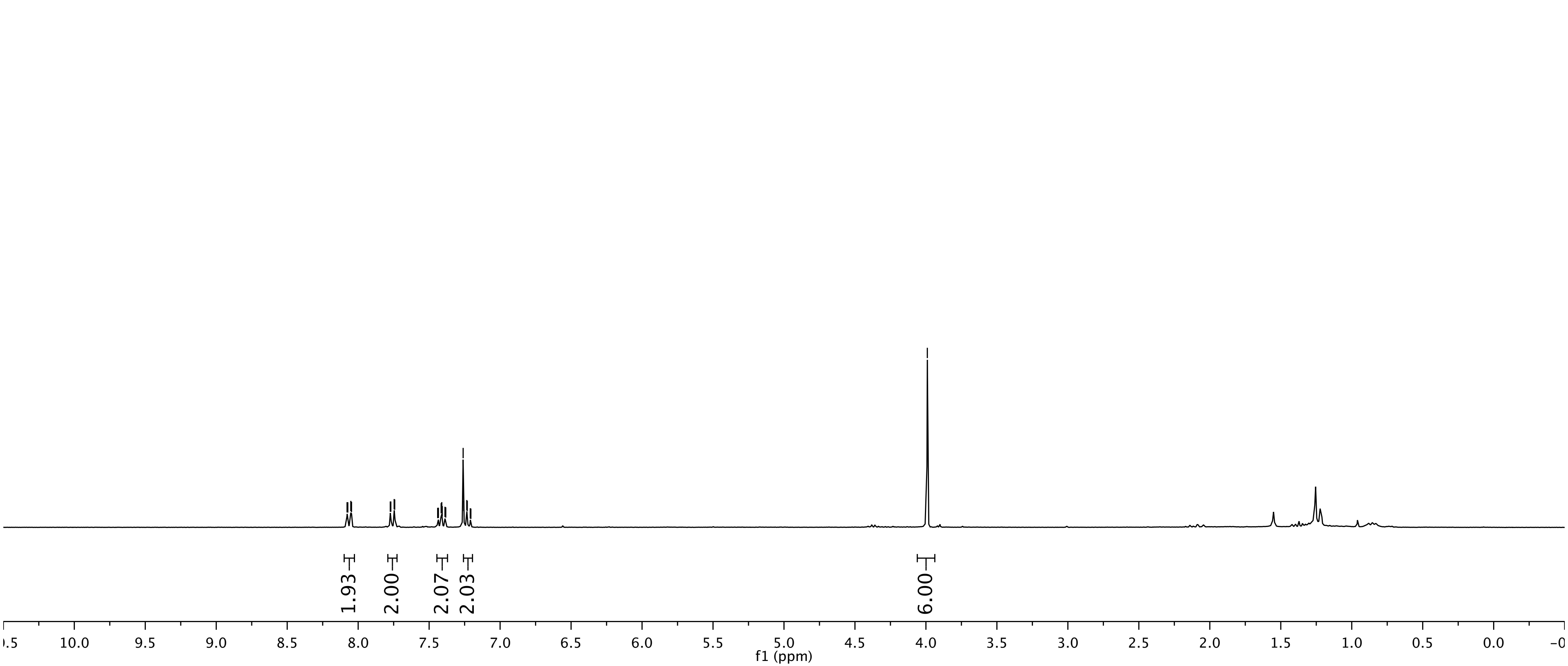
8.078
8.073
8.052
8.047
7.774
7.770
7.746
7.743
7.440
7.435
7.415
7.411
7.408
7.388
7.383
7.260
7.256
7.235
7.231
7.210
7.206
3.990
1.93
2.00
2.07
2.03
6.00
10.0
9.5
9.0
8.5
8.0
7.5
7.0
6.5
6.0
5.5
5.0
4.5
4.0
3.5
3.0
2.5
2.0
1.5
1.0
0.5
0.0
f1 (ppm)
